# Trajectory optimization for wall-building robots in accordance with nonlinear viscoelastic cement mortar environment

**DOI:** 10.1038/s41598-026-58844-w

**Published:** 2026-06-29

**Authors:** Qingyi Shi, Liu Cao, Chunyan Kong, Xiangtao Xiao, Ke Chen, Yiwei Fan, Jing Chang

**Affiliations:** 1 School of Mechanical Engineering, Xihua University, Chengdu, 610039 China; 2 CCTEG Chongqing Research Institute, Chongqing, 400039 China; 3 Anhui Engineering Research Center for High Efficiency Intelligent Photovoltaic Module, Chaohu University, Hefei, 230000 China

**Keywords:** Cement mortar contact environment, Kriging surrogate model, FEPSO algorithm, Trajectory optimization, Masonry quality, Engineering, Mathematics and computing

## Abstract

To address the issue of suboptimal masonry quality in wall-building robots operating within the viscoelastic contact environment of cement mortar, a multi-objective trajectory optimization method is proposed based on Kriging surrogate modeling and the Fractal Evolutionary Particle Swarm Optimization (FEPSO) algorithm in this paper. First, orthogonal experimental design is employed to obtain design variable values, with corresponding objective function values derived experimentally. A Kriging surrogate model linking the objective function to design variables is established to overcome the difficulty in constructing a viscoelastic mechanical model for cement mortar. Subsequently, integrating the Kriging surrogate model, a multi-objective trajectory optimization model for bricklaying is developed. The FEPSO algorithm is employed to solve this model, simultaneously enhancing masonry quality while optimizing other performance metrics. The FEPSO algorithm is compared with NSGA-II and MOPSO optimization algorithms, demonstrating its superiority. Then, the TOPSIS algorithm is applied to derive a compromise solution from the Pareto solution set, which is adopted as the optimal masonry scheme. Finally, the optimal masonry scheme is contrasted with the standard door-shaped trajectory planning method. Results indicate that trajectory optimization increased the wall-building robot’s efficiency by 23.66%, reduced energy consumption by 29.33%, and improved trajectory smoothness by 90.47%. Concurrently, environmental contact force decreased by 7.03%, and masonry error decreased from 2.57 to 0.14 mm. The proposed method enhances bricklaying quality and provides theoretical references and practical guidance for trajectory planning and construction quality control of similar intelligent construction robots.

## Introduction

The construction industry, as a pillar of the national economy, occupies a central position in the economic systems of numerous countries worldwide. By stimulating upstream and downstream industrial chains, creating employment opportunities, and driving technological innovation, this sector has become a vital engine propelling national GDP growth. Construction projects are inherently labor-intensive and inefficient, heavily reliant on manual processes that are prone to tool and human errors as well as fatigue^1^. Concurrently, the accelerated development of intelligent technologies provides the technical foundation for transforming traditional industries^2,3^. Against this backdrop, smart construction, leveraging its technological advantages, has emerged as a critical pathway for the construction industry to overcome developmental bottlenecks and achieve sustainable growth. It holds significant importance in propelling the sector’s transition from “labor-intensive” to “data-driven” operations^4,5^. In recent years, the field of construction automation has witnessed breakthrough developments in robotics technology. Currently, intelligent mobile mechanical systems with autonomous navigation capabilities and mechatronic devices are being progressively deployed on construction sites, enabling the precise execution of complex tasks such as such as concrete pouring, welding, spraying, and surveying. The application of construction robots has markedly enhanced construction efficiency and quality. Through precise automated operations, construction robots can complete large volumes of repetitive tasks in short timeframes, reducing human error and improving project quality. Simultaneously, construction robots can operate in high-risk environments, mitigating construction safety hazards^6^.

However, robots specifically designed for masonry wall construction remain uncommon. The primary reason lies in the traditional approach of using door-shaped trajectory planning for wall-building robots, which relies solely on positioning for wall construction. It does not account for the rheological parameter modeling of cement mortar. Cement mortar is a multi-phase porous composite composed of C-S-H hydration gels, unhydrated cement particles, fine aggregates, pore water and micro voids^7^. Its viscoelasticity originates from two aspects: the elastic response is provided by the rigid skeleton formed by aggregates and cement particles; the time-dependent viscous behavior is mainly caused by the viscous flow, sliding and rearrangement of flexible C-S-H gel networks, as well as the migration and seepage of free pore water under external load. Meanwhile, the gradual compaction of internal pores and the initiation and propagation of microcracks under increasing load lead to obvious strain-dependent and rate-dependent nonlinear characteristics. This inherent material nature makes mortar exhibit complex nonlinear viscoelastic responses during the bricklaying process. Moreover, under non-Newtonian fluid conditions, its path planning algorithm exhibits significant limitations in adaptive capability. These technical shortcomings directly result in quality issues during masonry operations, such as wall tilting and insufficient mortar joint saturation. Given these challenges, developing an intelligent trajectory planning method adapted to cement mortar environments has become a core research focus for enhancing the masonry techniques of wall-building robots.

To enhance the precision of robotic masonry techniques, the key lies in constructing a viscoelastic mechanical simulation model and coupling it with path planning algorithms. This ensures robotic arm stability during masonry operations under complex viscoelastic conditions. Currently, various viscoelastic constitutive models are widely adopted in engineering research. Sánchez et al.^8^ employed a generalized Maxwell viscoelastic model with Prony series in simulations to replicate the thermoforming process of polystyrene at temperatures corresponding to its crystalline melting point. Batt et al.^9^ investigated the free vibration and forced vibration responses of a linearly distributed parameter model for a viscoelastic rod with an externally attached tip mass, utilizing the classical Maxwell-Weichert model to characterize the viscoelastic system. Ruimin et al.^10^ derived the constitutive equations for natural sedimentary clays based on the Voigt model, providing a theoretical basis for finite element simulations and soil mechanical property testing. They also elucidated the fracture behavior of structural blocks through case studies. López-Almansa et al.^11^ employed the Kelvin-Voigt model to describe seismic impacts between adjacent structures and proposed a novel method for estimating its damping parameters. Hu et al.^12^ developed an analytical model for ground-deep circular tunnel interaction based on the viscoelastic Burgers model, validating its effectiveness by comparing results with numerical simulations. Overall, modeling approaches based on spatial geometry can yield analytical solutions, which play a crucial role in dealing with viscoelastic issues. However, the effectiveness of diverse viscoelastic models varies, establishing model relationships is relatively complex, and the structural characteristics of the models themselves make it hard to determine accurate outcomes. Furthermore, establishing their mechanical models requires knowledge of relevant moduli such as relaxation modulus and creep flexibility, yet current research on cement mortar material properties remains limited^13,14^. Consequently, the viscoelastic nature of cement mortar necessitates additional research, hindering the accurate modeling of mechanical response of bricklaying process using particular viscoelastic frameworks.

In optimization, Kriging surrogate models can smoothly fit the original objective function with fewer sample data points. By selecting different regression models and correlation functions, they flexibly adapt to varying trends in objective functions while covering inherent uncertainties. This makes Kriging an effective modeling approach for addressing computational overload, slow convergence, and high uncertainty in optimization design. Kriging demonstrates exceptional flexibility and robustness when approximating complex multidimensional functions, having been applied to model responses in numerous engineering systems. The U.S. Argonne National Laboratory^15^ and Massachusetts Institute of Technology^16^ employed Kriging surrogate models to optimize core parameters and geometric dimensions for liquid metal reactors and high-flux research reactors, respectively. Inha University in South Korea employed a Kriging surrogate model to optimize the geometry of the inlet pressure chamber in an air-cooled reactor^17^. Huang et al.^18^ explored a Kriging model for constructing response surfaces to rapidly evaluate the activity of key radionuclides in spent nuclear fuel across varying cooling times. Zhou et al.^19^ constructed Kriging models to predict compressor blade positional errors and torsional errors, analyzing the influence of machining parameters on these errors. The established models achieved prediction accuracies exceeding 0.85, providing robust support for optimizing compressor blade manufacturing processes. The above studies demonstrate that Kriging surrogate models offer high prediction accuracy and computational efficiency.

Masonry cement mortar exhibits complex nonlinear viscoelastic behavior under construction loads. Classic analytical rheological constitutive models are primarily developed under ideal laboratory conditions and require extensive experimental data for parameter calibration, making them inadequate for modeling the dynamic, multi-load coupled conditions encountered during field masonry applications. The primary objective of this study is to analyze the macroscopic mechanical responses of mortar under construction loads tailored to actual masonry processes, rather than conducting fundamental rheological mechanism studies on the material itself. The Kriging model, as a high-precision surrogate model, eliminates the need for predefined physical assumptions about constitutive relationships and enables direct establishment of correlations between operational parameters and mechanical responses based on experimental data. This study trains the model using experimental data obtained from actual masonry load paths and on-site conditions. The model implicitly incorporates all nonlinear viscoelastic characteristics of mortar, including viscoelastic hysteresis, strain rate effects, and stress relaxation, and is capable of reproducing the overall mechanical behavior of mortar under construction loads. Combined with optimization methods, Kriging surrogate models are feasible and effective for constructing mechanical models of cement mortar.

Reasonable trajectory planning is crucial for ensuring the accuracy and efficiency of wall-building robot movements^20^. It directly impacts the robot’s operational performance, safety, and practicality. A well-designed trajectory planning system can reduce energy consumption, shorten task duration, and prevent unnecessary environmental damage^21^. Over the past few years, trajectory planning has become a key area of interest among researchers. Ekrem et al.^22^ made use of a 6-DOF robotic arm provided by the Mechatronics Engineering Laboratory at Isparta University of Applied Sciences. They utilized the Particle Swarm Optimization (PSO) algorithm for trajectory planning, achieving time optimization by selecting the shortest path between two points. Wang et al.^23^ centered on a metamorphic palletizing robot. The main objective is to tackle the intricate problem of multi-objective trajectory planning during the robot’s movement, emphasizing the reduction of time, energy usage, and jerk. The optimization procedure utilizes the Non-dominated Sorting Genetic Algorithm with an elite strategy (NSGA-II), and Particle Swarm Optimization (PSO) is incorporated into the optimization workflow to detect specific metamorphic points. Yagüe et al.^24^ introduced a innovative human-guided trajectory optimization algorithm. It effectively combines human expertise, Particle Swarm Optimization (PSO), and Pattern Search (PS). The outcomes show that this method significantly boosts robotic system efficiency, with cycle time improvements of up to 16.69% compared to trajectories designed by experts. Elgohr et al.^25^ pioneered the Whale Genetic Algorithm (WGA), merging the global search proficiency of the Whale Optimization Algorithm (WOA) with GA’s local refinement strengths. WGA focuses on robotic arm path optimization by minimizing both temporal reachability and energy expenditure while maintaining kinematic feasibility and operational boundary conditions. A time-optimized trajectory planning approach for metamorphic industrial robots is introduced. These studies addressed trajectory planning for robotic arms in motion spaces, successfully optimizing movement paths to achieve their respective objectives.

However, the aforementioned research did not address contact issues with uncertain viscoelastic environments. Cement mortar behaves as a non-deterministic viscoelastic substance. The porous microstructure of the mortar matrix, combined with the interfacial transition zones between cement paste and aggregates, significantly complicates its material properties^26^. Current research on wall construction robots and intelligent construction optimization primarily focuses on path planning, motion control, and construction efficiency improvement. Mainstream methods include A*, genetic algorithms, particle swarm optimization^27,28^, and RRT*, all aiming to reduce path length, minimize idle travel distances, and enhance construction speed. Existing approaches generally treat cement mortar as a linear elastic material, neglecting its nonlinear viscoelastic properties and time-dependent mechanical characteristics, thereby failing to accurately reflect material behavior and structural stress responses during actual construction. Moreover, most optimization methods address single objectives, while only a few multi-objective approaches employ weighting mechanisms, unable to generate Pareto optimal solutions that balance mechanical performance, energy consumption, efficiency, and construction quality. Additionally, issues such as disconnects between agent models, optimization algorithms, and decision-making processes, as well as decoupling between path planning and structural mechanical responses, hinder the fulfillment of high-performance intelligent construction requirements. Unlike conventional trajectory optimization problems, robotic bricklaying involves contact with uncertain viscoelastic materials like cement mortar. Moreover, robotic arm trajectory optimization encompasses multiple objectives including efficiency, energy consumption, smoothness, applied forces, and precision. This constitutes a complex, high-dimensional, nonlinear multi-objective optimization problem, rendering traditional trajectory planning methods ill-suited for uncertain viscoelastic environments like cement mortar. Building upon traditional particle swarm optimization, a Fractal Evolutionary Particle Swarm Optimization (FEPSO) algorithm was proposed^29^. FEPSO leverages the random motion characteristics of fractal Brownian motion (fBm) to simulate the unknown properties of the objective function, while implicit trend changes mimic general tendency of extremum variations in the objective function. This approach overcomes issues of excessive random evolution and premature convergence in individual particles. During the fractal evolution phase, particles traverse the solution subspace via fBm with distinct fractal parameters, updating their components accordingly. Compared to conventional trajectory optimization algorithms, this approach demonstrates superior global search capabilities, effectively addressing the challenges of optimizing complex, high-dimensional nonlinear multi-objective functions.

Inspired by this, addressing the challenges that existing viscoelastic models struggle to construct for cement mortar and existing trajectory planning techniques struggle to accommodate the unpredictable viscoelastic behavior of cement mortar, this paper proposes an integrated optimization framework combining Kriging, FEPSO, and TOPSIS, achieving innovative optimization approaches by accounting for the nonlinear mechanical characteristics of cement mortar. The Kriging surrogate model provides high-precision fitting of nonlinear viscoelastic responses, reducing computational costs associated with multi-field coupling; the FEPSO algorithm enhances global search capability, yielding a uniformly distributed set of Pareto optimal solutions; TOPSIS is employed to facilitate multi-objective decision-making, completing a closed-loop process spanning approximate modeling, multi-objective optimization, and engineering selection. This method overcomes limitations imposed by traditional linear elasticity assumptions, effectively addresses the nonlinear viscoelastic behavior of cement mortar, and achieves synergistic optimization of construction paths and structural mechanical performance. It offers a more practical optimization approach for wall-building robots, demonstrating significant superiority over existing methods in computational efficiency, optimization accuracy, and multi-objective balancing capabilities. The key contributions include:

1. An experimental testing environment for autonomous masonry robots was developed, introducing an innovative trajectory optimization approach that combines Kriging modeling with the FEPSO methodology. Compared with previous research on robot trajectory optimization, this method fully accounts for the viscoelastic properties of cement mortar and incorporates a dedicated viscoelastic model into the optimization process, thereby establishing a novel trajectory optimization approach tailored for cement mortar environments.
2. Critical design factors, performance metrics, and limitations were systematically defined. A curated dataset was formulated to develop a Kriging surrogate model for cement mortar, elucidating the correlations between input parameters and output objectives. This addresses the issue of traditional space-based geometric approaches to viscoelastic modeling being ill-suited for constructing viscoelastic models of cement mortar used in brick masonry processes.
3. A mathematical model for optimizing wall-building robot trajectories was developed. The solution was obtained using the Fractal Evolutionary Particle Swarm Optimization algorithm, which was compared with NSGA-II and MOPSO optimization algorithms. The NSGA-II and MOPSO algorithms are not suitable for optimizing bricklaying processes, demonstrating the unique advantages of the FEPSO algorithm in solving optimization problems with irregularly shaped Pareto solution sets.
4. The TOPSIS methodology was utilized to determine the most favorable masonry configuration within the Pareto-optimal solution set. Comparative analysis was conducted against conventional door-shaped trajectory planning techniques, thereby substantiating the efficacy of the introduced trajectory optimization approach. Compared with traditional portal trajectory planning techniques, the proposed method not only optimizes the robot’s joint space trajectories, angular velocity, and acceleration profiles, but also reduces energy consumption and contact forces of the masonry robot, thereby enhancing both construction efficiency and precision.

The subsequent discourse is systematically organized. Section "Trajectory planning for wall-building robots" delineates the robotic masonry platform, introduces the advanced trajectory optimization technique, and outlines the experimental procedures. Section "Construction of Kriging surrogate models" establishes the Kriging approximation framework. Section "Trajectory optimization" establishes the mathematical model for trajectory optimization and introduces the FEPSO algorithm. Section "Results and discussion" validates the Kriging model and performs trajectory optimization. Finally, Section "Conclusion" presents the conclusions.

## Trajectory planning for wall-building robots

### Trajectory planning

**Fig. 1:**
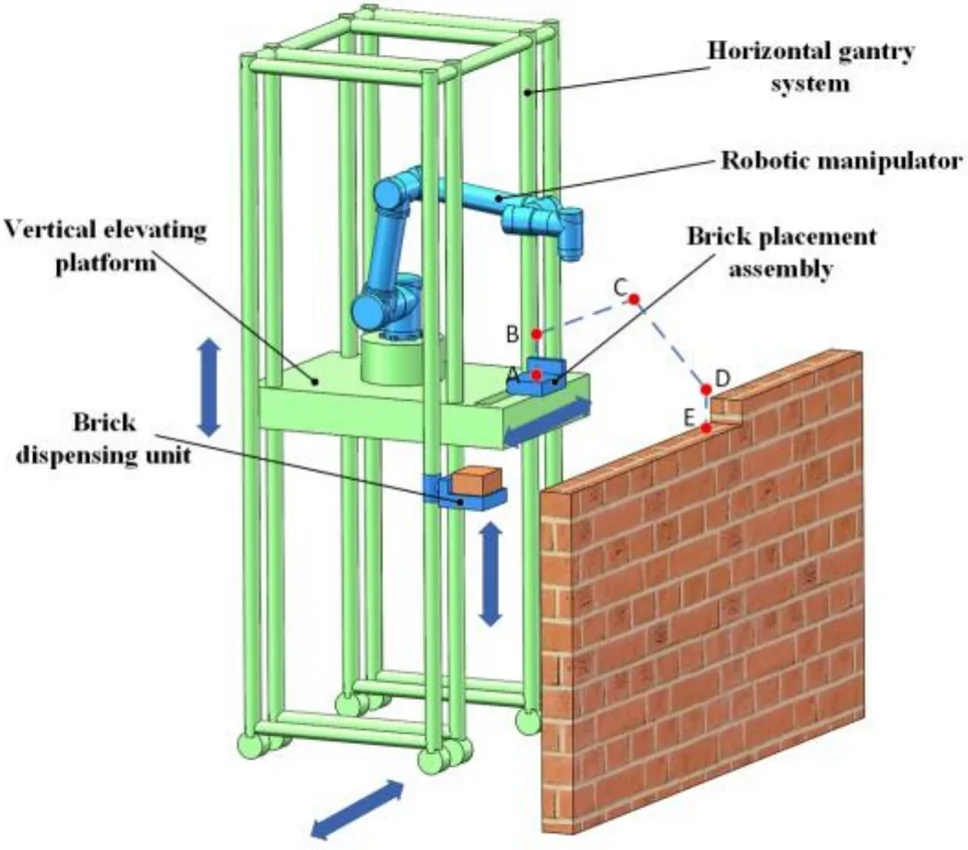
Conceptual 3D prototype of a wall-building robot.

Figure 1 depicts the conceptual 3D prototype of our wall-building robot, primarily illustrating its design intent to perform core bricklaying functions. It is conceptual figure of the wall-building robot. Detailed structural specifications require separate engineering. The robotic masonry platform consists of five primary components: articulated robotic manipulator, brick dispensing unit, brick placement assembly, vertical elevating platform, and horizontal gantry system. The dispensing and placement units regulate brick material flow, the elevating platform controls z-axis motion, the gantry system enables xy-plane displacement, while the robotic manipulator executes precise brick grasping and placement functions.

**Fig. 2:**
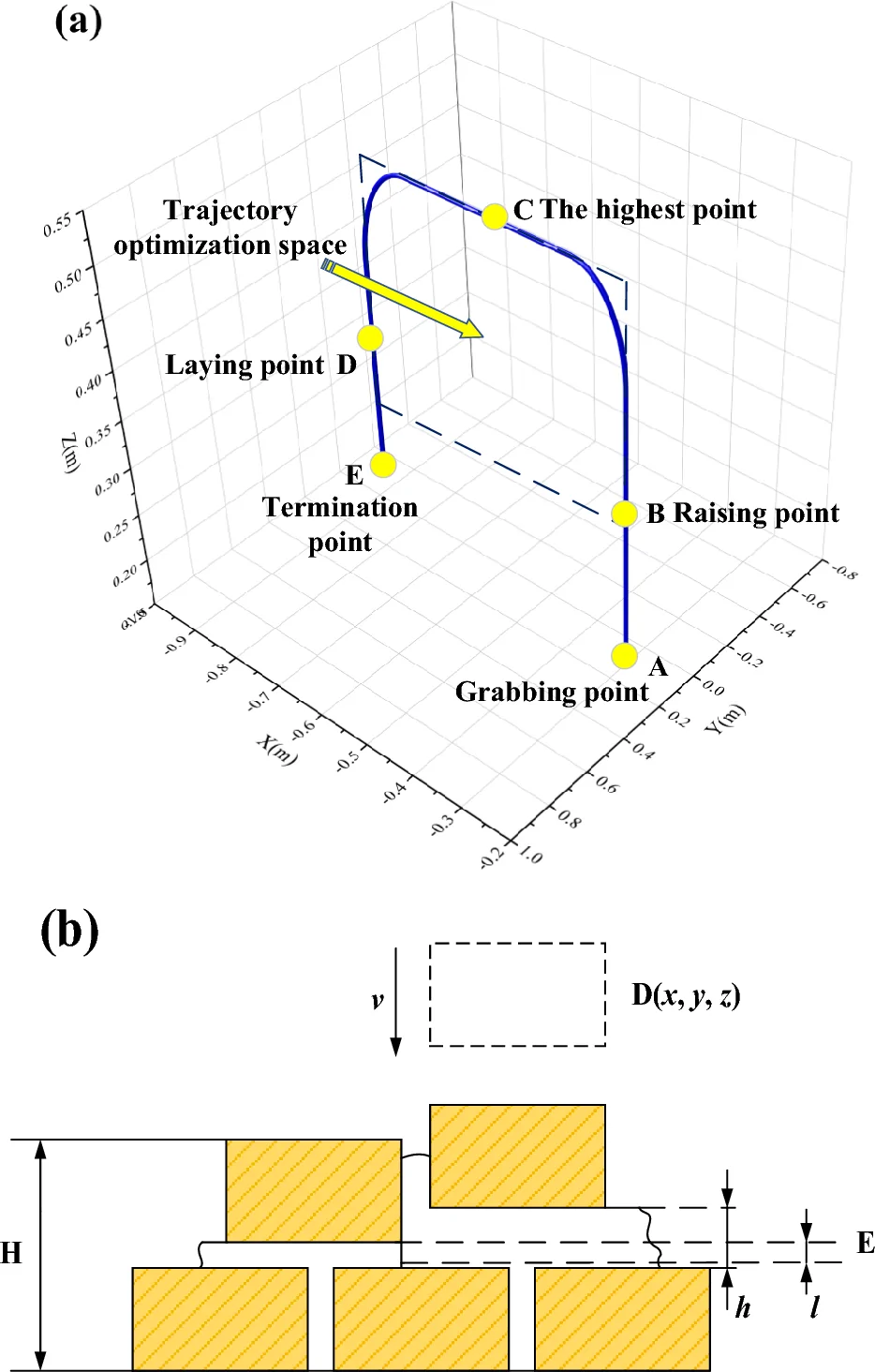
Traditional door-shaped masonry trajectory and brick geometric placement framework. (**a**) schematic representation of door-shaped motion trajectory. (**b**) geometrical framework of brick placement.

The masonry task performed by wall-building robots for large structures such as buildings involves single-rule bricks and non-Newtonian fluid cement mortar coming into contact and bonding at designated positions under external force. Traditional wall-building robots primarily operate through teach-in methods, typically following the “door-shaped” trajectory shown in Fig. 2a. The robot initiates brick pickup at point A, then executes linear motion. Upon reaching the height of point C, the brick undergoes horizontal linear movement, aligns above the placement position, and finally deposits vertically at the designated location. To achieve smoother motion, two-segment circular arc trajectories are occasionally employed for transitions. However, existing masonry techniques and robotic systems cannot overcome the nonlinear viscoelastic contact and damping forces caused by the non-Newtonian fluid properties of mortar during single-task masonry. This impedes trajectory planning for wall-building robots, making it difficult to achieve high-precision masonry requirements. Consequently, brick placement errors commonly exceed 1 mm. The precision of brick laying directly impacts the quality of building construction. Substandard laying techniques result in uneven walls, poor load-bearing capacity, low compressive strength, and may even cause building collapse and safety incidents. Notably, the frequency-dependent viscoelastic coupling effects manifest as a nonholonomic constraint in the robot’s operational space, fundamentally limiting the applicability of analytical optimization frameworks. This hinders the achievement of high optimization and efficiency in optimization strategies. Therefore, considering the viscoelastic characteristics of cement mortar, developing a trajectory optimization technique suitable for bricklaying is crucial for enhancing the motion stability, laying efficiency, and precision of wall-building robots. This represents a key prerequisite for advancing the intelligent automation of bricklaying operations.

The trajectory optimization framework in this work partitions the full trajectory into two distinct phases: a free-motion phase (ABCD segment) and a bricklaying phase (DE segment). Critical path points (ABCE) are predefined, whereas point D acts as a variable node, whose kinematic parameters are resolved through free-motion optimization. For computational efficiency, the DE segment assumes constant-velocity motion. During free-motion execution, segment AB follows linear interpolation, while BCD forms the optimization domain. The dotted bounding box in Fig. 2a defines the permissible workspace for end-effector trajectory generation.

The wall-building robot’s geometric placement framework is shown in Fig. 2b, where H designates the target height post-placement, D identifies the brick’s designated lay position, *h* corresponds to the cement mortar thickness, E defines the nominal stopping point, *l* adjusts for positional deviation, and *v* denotes the operational speed during placement. As depicted in Fig. 2b, conventional wall-building robots depend exclusively on positioning accuracy for brick placement, releasing the brick upon reaching point E. However, during placement, the brick contacts the mortar, encountering significant viscoelastic damping from the mortar, preventing precise positioning at the target location. Therefore, to address this issue, we added a small distance *l* at point E. This allows the robot to move slightly beyond point E, thereby counteracting the viscoelastic damping effect of the mortar. This approach mimics the pressing action a human worker would perform during bricklaying to achieve greater positional accuracy.

**Fig. 3:**
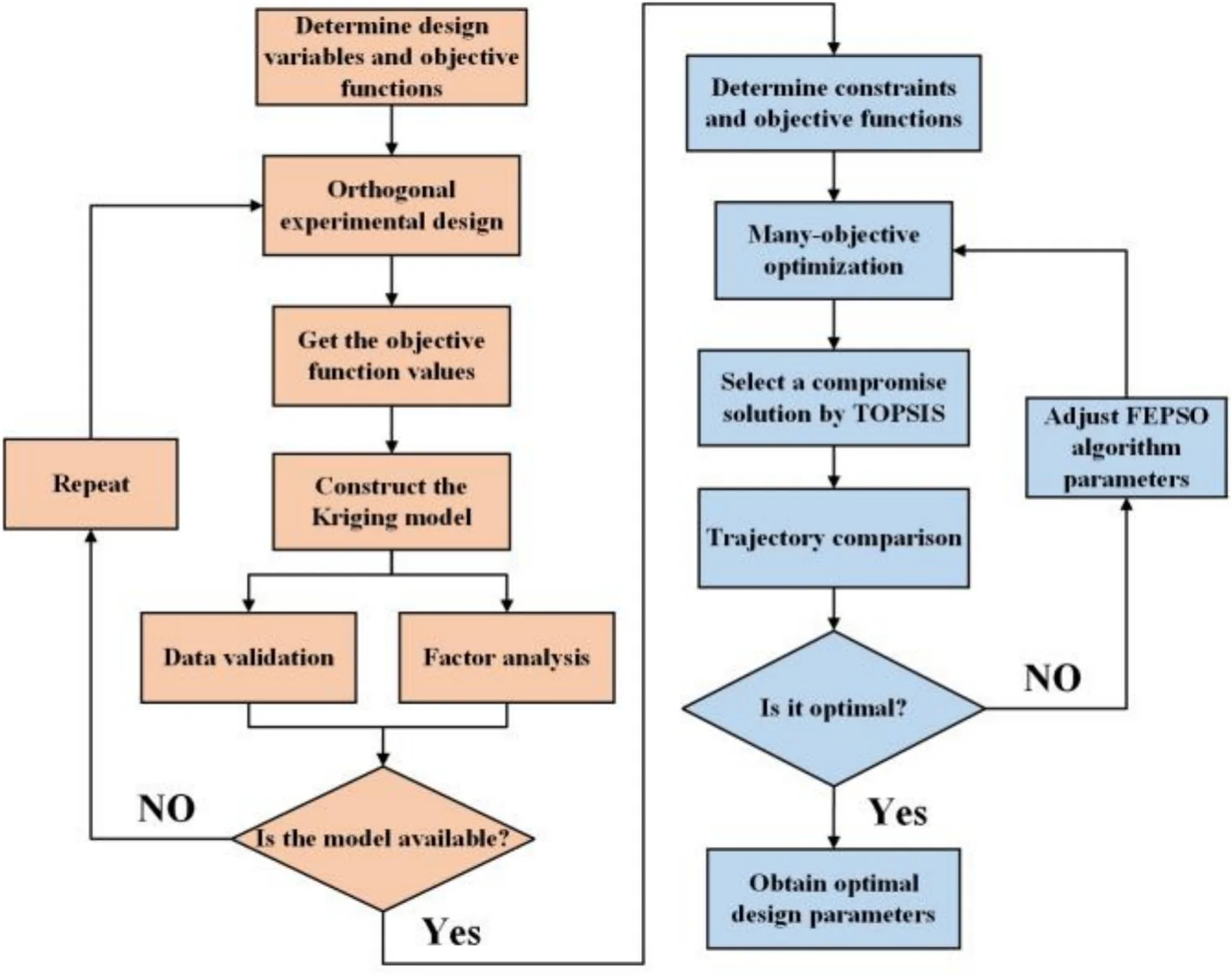
Flowchart of the suggested trajectory optimization methodology.

To investigate the relationship between robotic motion parameters, bricklaying models, and bricklaying accuracy metrics, and to identify optimal bricklaying parameters, the optimization methodology suggested in the current work is illustrated in Fig. 3. By treating the position and velocity of laying point D, position compensation, and mortar application thickness as design variables, and using the maximum contact force during brick laying and brick laying error as target response functions, an orthogonal experimental design method is employed to sample the design variables, forming an experimental plan. Experimental data are then used to obtain the values of the target functions. Subsequently, a Kriging surrogate model is established between the target functions and the design variables. Subsequently, a mathematical model for optimizing the second track segment (ABCD segment) is established. Finally, by integrating the Kriging surrogate model, a trajectory optimization model for the ABCDE section is constructed. The FEPSO technique is leveraged to obtain the Pareto solution set of the design parameters for the ABCDE section, and the TOPSIS algorithm is employed to find the compromise solution within the Pareto solution set. This compromise solution can minimize the objective response function values, thereby determining the optimal bricklaying scheme.

### Nova5 robotic arm

**Fig. 4:**
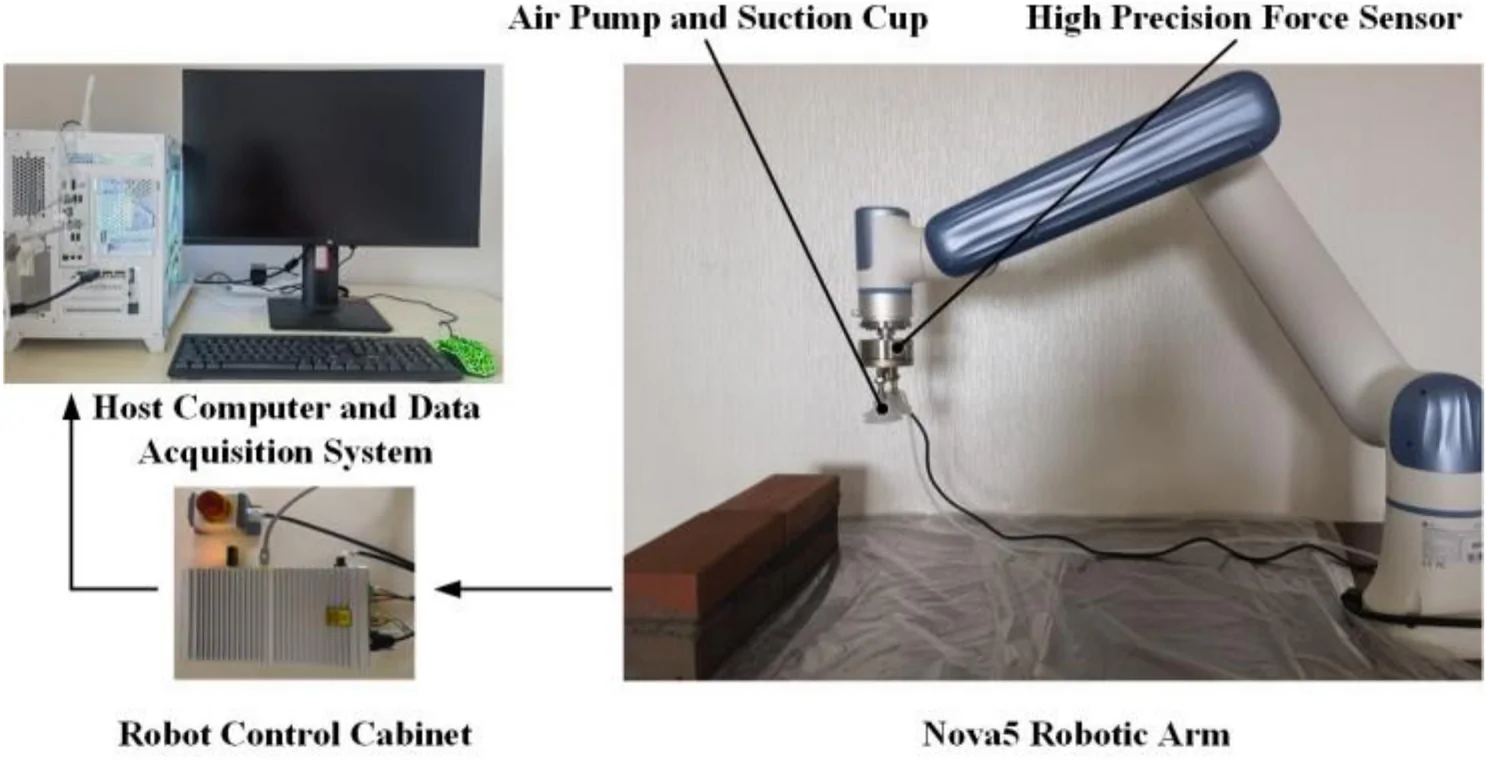
Wall-building robot experimental setup.

The experimental verification system for the wall-building robot, illustrated in Fig. 4, simulates the bricklaying process performed by the robotic arm. It primarily consists of hardware and software components including the robotic arm, host computer, data acquisition system, and high-precision force sensing system. The robotic arm utilizes the DOBOT Nova5 from Yuejiang Robotics, designed to grasp bricks and execute bricklaying tasks. The high-precision force sensor at the robotic arm’s end effector primarily records the magnitude of applied contact forces. The host computer system can drive the robotic arm’s movements via the robot control cabinet and collect various motion parameters during bricklaying tasks.

The Denavit-Hartenberg parameters for the Nova5 robotic arm are listed in Table 1. Following the modified DH framework, the kinematic model of the robot was derived. The homogeneous transformation matrix between consecutive links is defined by:

$${}_{i}{}^{i-1}T=\mathrm{R}\mathrm{o}\mathrm{t}\left(X,{\alpha}_{i-1}\right)\mathrm{T}\mathrm{r}\mathrm{a}\mathrm{n}\mathrm{s}\left(X,{a}_{i-1}\right)\mathrm{R}\mathrm{o}\mathrm{t}\left(Z,{q}_{i}\right)\mathrm{T}\mathrm{r}\mathrm{a}\mathrm{n}\mathrm{s}\left(Z,{d}_{i}\right)=$$

1 $$\left[\begin{array}{c}cos{q}_{i}\\ sin{q}_{i}cos{\alpha}_{i-1}\\ sin{q}_{i}sin{\alpha}_{i-1}\\ 0\end{array} \begin{array}{c}-sin{q}_{i}\\ cos{q}_{i}cos{\alpha}_{i-1}\\ cos{q}_{i}sin{\alpha}_{i-1}\\ 0\end{array} \begin{array}{c}0\\ -sin{\alpha}_{i-1}\\ cos{\alpha}_{i-1}\\ 0\end{array} \begin{array}{c}{a}_{i-1}\\ -sin{\alpha}_{i-1}\cdot {d}_{i}\\ cos{\alpha}_{i-1}\cdot {d}_{i}\\ 1\end{array}\right]$$

where ${q}_{i}$, ${d}_{i}$, ${\alpha}_{i}$, and ${a}_{i}$ represent the joint angle, link offset, link length, and link twist angle, respectively. $Rot\left(X,{\alpha}_{i-1}\right)$ denotes the rotation angle ${\alpha}_{i-1}$ about the X-axis; $Trans\left(X,{a}_{i-1}\right)$ denotes the translation distance ${a}_{i-1}$ along the X-axis; $Rot\left(Z,{q}_{i}\right)$ denotes the rotation angle ${q}_{i}$ about the Z-axis; $Trans\left(Z,{d}_{i}\right)$ denotes the translation distance ${d}_{i}$ along the Z-axis.

**Table 1:** DH kinematic parameters of Nova5 robotic arm.

Kinematics Parameters	$\alpha /^\circ$	*d/mm*	$q/^\circ$	*a/mm*
Joint1	0	${d}_{1}=0.240$	${q}_{1}$	0
Joint2	90	0	${q}_{2}$	0
Joint3	0	0	${q}_{3}$	${a}_{2}=-\hspace{0.17em}0.400$
Joint4	0	${d}_{4}=0.135$	${q}_{4}$	${a}_{3}=-\hspace{0.17em}0.330$
Joint5	90	${d}_{5}=0.120$	${q}_{5}$	0
Joint6	-90	${d}_{6}=0.088$	${q}_{6}$	0

The robot’s kinematic model as shown in Eq. (2) can be derived through matrix multiplication of the link transformation matrices.

2 $${}_{6}{}^{0}T={}_{1}{}^{0}T{}_{2}{}^{1}T{}_{3}{}^{2}T{}_{4}{}^{3}T{}_{5}{}^{4}T{}_{6}{}^{5}T$$

This experimental research project primarily consists of two parts: constructing a Kriging-based response surface approximations for design parameter- performance relationships, and conducting trajectory optimization experiments for the masonry process. This study used cement mortar with a fixed mix ratio, consisting of P・O 42.5 ordinary Portland cement, medium-grade sand aggregate with a particle size range of 0.16–2.36 mm, a water-cement ratio of 0.45, and a cement-to-sand mass ratio of 1:3. The mortar parameters were kept constant throughout the entire testing process. The typical mechanical properties of the cement mortar used in this study are as follows: elastic modulus E = 12.5 ± 1.5 GPa; equivalent viscoelastic modulus decaying from 10–15 to 2–4 GPa; initial stress relaxation modulus of 14.2 GPa, long-term modulus of 3.1 GPa, and characteristic relaxation time of approximately 25 min; Poisson’s ratio is approximately 0.2, and density is approximately 2.0 g/cm^3^. The masonry bricks used are standard MU10 fired bricks, with dimensions of 200 mm × 100 mm × 48 mm, and all physical properties are uniform. The test and curing environment was controlled at a temperature of 20 ± 2 °C and relative humidity of 60% ± 5%, using standard film-covered moisture-retaining curing. With all baseline conditions held constant and only the target variables altered, the experimental design was standardized, ensuring good reproducibility of the results. Based on the research objectives, the following experimental plan was designed:

1. The experimental design incorporated four controlled factors: Point D’s kinematic parameters (position/velocity), Point E’s positional compensation, and mortar application thickness, with orthogonal sampling used to generate the test configuration.
2. Mix cement mortar and allow 5 min curing before application. Configure Point D’s spatial coordinates and motion parameters, along with Point E’s positional offsets, based on predetermined sampling values. Proceed to install bricks at designated reference Point A. The robotic arm follows the preset motion trajectory, guiding the end-effector to perform vertical bricklaying. Motion data is measured via the robot’s high-precision encoders, while external force data is captured by a 6-axis force sensor mounted at the end-effector’s front. Measured data is extracted through the data acquisition system.
3. Following brick installation, initiate robotic arm retraction. Utilize vernier calipers to obtain dimensional readings from all four brick corners. Compute the laid height parameter by deriving the square root of the arithmetic mean of the squared deviations from these four measurement points. Determine the laying error for each experiment by subtracting the standard laid height from the measured brick height.
4. Construct a Kriging surrogate model for the cement mortar using the measured dataset. Investigate the impact of every parametric factor upon the target performance metric and verify the precision of the nonlinear viscoelastic contact mechanics simulation framework.
5. Integrating the Kriging approximation technique, construct a computational framework for trajectory optimization of the ABCDE segment and implement its solution through the FEPSO evolutionary algorithm. Upon deriving the optimal trajectory parameters, recalibrate the system and execute experimental trials. Benchmark these outcomes against conventional door-shaped masonry techniques to demonstrate the enhanced performance characteristics of the proposed methodology.

## Construction of Kriging surrogate models

### Kriging surrogate models

The use of Kriging models has gained increasing popularity due to their flexibility in approximating various complex response functions. As a statistical linear regression-based interpolation technique^30^, Kriging models predict information at a specific point by estimating the response value through a weighted linear combination of information from the neighborhood of the target point. The model incorporates a regression function and a random variable conforming to specific predefined statistical patterns. Recognized as an optimal linear unbiased estimator, we employ the Kriging model to construct a viscoelastic mechanical model for cement mortar. This study employs the Kriging method to construct an agent model, using the Gaussian kernel as the associated kernel function. This kernel exhibits excellent smoothness and strong capability in fitting nonlinear responses, making it the most widely adopted kernel function in engineering agent models. Its mathematical expression is as follows:

3 $${\boldsymbol{R}}\left({\boldsymbol{\theta}},{\boldsymbol{s}}\right)=\mathbf{e}\mathbf{x}\mathbf{p}\left(-\sum_{{\boldsymbol{i}}=1}^{{\boldsymbol{n}}}{{\boldsymbol{\theta}}}_{{\boldsymbol{i}}}{\left|{{\boldsymbol{s}}}_{{\boldsymbol{i}}}-{{\boldsymbol{x}}}_{{\boldsymbol{i}}}\right|}^{2}\right)$$

In the formula: ${\boldsymbol{\theta}}$ denotes the parameter to be estimated, while ${\boldsymbol{s}}$ and ${\boldsymbol{x}}$ represent any two sets of sample input vectors.

### Design variables and objective response function

The primary performance metric of interest is the masonry precision of bricks, which directly determines the construction quality of a wall and even the structural integrity of a building. Therefore, we designate it as one of the objective functions requiring optimization. The masonry precision manifests as masonry error, which we characterize using Eq. (4). The deviation is quantified by comparing the standard masonry height with the measured height obtained from the brick’s four corner positions, with smaller deviations indicating superior construction accuracy. During bricklaying operations, the robotic arm interacts with the cement mortar’s viscoelastic medium, producing reaction forces proportional to the mortar’s compression level. Excessive reaction forces sustained over extended periods accelerate wear on robotic joints, shortening operational lifespan. Notably, monitoring these reaction forces provides indirect measurement of mortar compaction, which directly impacts construction quality. Consequently, the peak reaction force Fmax experienced by the robotic arm is designated as a critical optimization objective within our formulation. This force is directly measured by sensors at the robotic arm’s end effector.

4 $$\mathrm{E}\mathrm{r}\mathrm{r}\mathrm{o}\mathrm{r}=\sqrt{\frac{1}{4}{\sum}_{i=1}^{4}{H}_{i}^{2}}-{H}_{0}$$

where ${H}_{i}$ represents the measured values of the brick’s four corners in mm. ${H}_{0}$ represents the standard height for bricklaying, set at 106.31 mm in this study.

As illustrated in Fig. 2b, during the brick’s transition to the designated endpoint E, the operational duration is jointly determined by the elevation of point D and the brick’s translational speed *v*. To enhance robotic construction efficiency during the masonry process, both the spatial coordinates of point D and the translational velocity *v* are identified as key design parameters. This optimization framework incorporates a previously defined positional compensation variable *l*, which remains a critical factor in the objective function formulation. Thus, we include it as a design variable. Additionally, the viscoelastic resistance experienced by the brick varies with different mortar application thicknesses *h*, potentially affecting the laying outcome. Consequently, we also incorporate mortar thickness as a design variable.

During masonry operations, the design parameters and objective response metrics are strictly governed by construction specifications. When the maximum contact force exceeds 9500 g, excessive environmental interaction between the robotic arm and its surroundings may induce mechanical oscillations. This not only affects bricklaying quality but also adversely impacts the robotic arm’s lifespan. Therefore, constraints are imposed on *l*, *h*, *v*, and Fmax. Furthermore, as shown in Eq. (3), when the masonry error is less than zero, a smaller error indicates lower masonry precision. Consequently, scenarios with negative masonry errors are excluded from consideration. Furthermore, the elevation of point D must remain within reasonable limits, necessitating the imposition of positional constraints. Empirical findings from experimental trials have established the permissible ranges and boundary conditions for the design variables, as detailed in Table 2.

**Table 2:** Design variables and constraints.

limits	Design variables				Objective functions	
	$x\left(mm\right)$	$h\left(mm\right)$	$l\left(mm\right)$	$v\left(mm/s\right)$	$Fmax\left(g\right)$	$Error\left(mm\right)$
Minimum	252	10	0.1	10	–	0
Maximum	400	30	10	150	9500	–

### Establishing the Kriging model

Establishing a Kriging model first requires designing the sample point layout. Orthogonal experimental design (OED) is an experimental arrangement method based on orthogonal arrays, suitable for multi-factor, multi-level research scenarios. Its core principle involves selecting representative level combinations through orthogonality to ensure experimental points exhibit “uniform dispersion and uniform comparability.” This approach reduces the number of experiments while maintaining comprehensive and reliable results^31^. Consequently, this research adopts an orthogonal experimental design methodology. Based on the design parameters listed in Table 2, this study employed a four-factor, three-level orthogonal experiment for sample selection, resulting in a total of 18 valid experimental samples (sample size N = 18). The four design variables—*x*, *h*, *l*, and *v*—are all continuous independent variables, with the following ranges: variable *x*∈[252, 400]; Variable *l*∈[0.1, 10]; variable *v*∈[10, 150]; variable *h*∈^10,30^. All samples were arranged strictly according to the orthogonal table, covering the full range of values for each variable to ensure the uniformity and representativeness of the sample space. Each orthogonal experiment was repeated three times, and the average value was taken as the value of the objective function. The experimental process strictly controlled environmental conditions, equipment, and operational procedures to ensure consistency and avoid systematic errors. The 3σ criterion was used to test for outliers in the samples, and outlier detection was performed separately for the objective functions Fmax and Error. No outliers were detected in the 18 sets of samples, and all samples were retained for modeling. The objective function values obtained from these experiments constitute the initial sample dataset; the complete results are shown in Table 3. Subsequently, both the design variables and corresponding objective function outputs were incorporated into the Kriging surrogate model to construct their mathematical relationship. Building upon this established framework, the multi-objective optimization challenge for the DE segment can be formally articulated as:

5 $$\left\{ {\begin{array}{*{20}l} {{\mathrm{Minimize}}:} \hfill & {{\mathrm{Error}} = f_{1} \left( {x,{ }l,{ }v,{ }h} \right)} \hfill \\ {{\mathrm{Minimize}}:} \hfill & {{\mathrm{Fmax}} = f_{2} \left( {x,{ }l,{ }v,{ }h} \right)} \hfill \\ {} \hfill & {252 \le x \le 400} \hfill \\ {} \hfill & {0.1 \le l \le 10} \hfill \\ {{\text{Subject to}}:} \hfill & {10 \le v \le 150} \hfill \\ {} \hfill & {10 \le h \le 30} \hfill \\ {} \hfill & {f_{1} > 0} \hfill \\ {} \hfill & {f_{2} < 9500} \hfill \\ \end{array} } \right.$$

**Table 3:** Orthogonal test design and corresponding results.

Number	Design variables				Objective functions	
	$x\left(mm\right)$	$l\left(mm\right)$	$v\left(mm\right)$	$h\left(mm/s\right)$	$Fmax\left(g\right)$	$Error\left(mm\right)$
1	252	0.1	10	10	6564.37	− 0.02
2	252	5.05	80	10	7219.28	− 2.35
3	326	0.1	150	10	7257.49	− 2.62
4	326	10	10	10	7221.37	− 2.86
5	400	5.05	150	10	7459.05	− 3.68
6	400	10	80	10	7468.49	− 3.62
7	252	0.1	150	20	8176.76	0.51
8	252	10	10	20	8210.55	0.13
9	326	5.05	80	20	8192.58	1.26
10	326	10	150	20	8939.96	− 0.52
11	400	0.1	80	20	7831.17	1.97
12	400	5.05	10	20	7810.02	1.62
13	252	5.05	150	30	9518.71	3.32
14	252	10	80	30	9514.96	3.23
15	326	0.1	80	30	8709.14	4.30
16	326	5.05	10	30	8723.94	4.08
17	400	0.1	10	30	8148.32	4.61
18	400	10	150	30	9889.96	3.04

## Trajectory optimization

### B-spline trajectory planning

The joint-space trajectory of the wall-building robot is planned using seventh-order non-uniform B-spline curves. To improve computational efficiency, the De-Boor algorithm is employed to derive the recursive formula for B-spline curves as follows:

6 $$\left\{ {\begin{array}{*{20}l} {p\left( u \right) = \mathop \sum \limits_{i = 0}^{n} d_{i} N_{i,k} \left( u \right) = \mathop \sum \limits_{j = i - k + 1}^{i} d_{j}^{l} N_{j,k - 1} \left( u \right) = \cdots = d_{i}^{k} ,{ }u_{i} \le u \le u_{i + 1} } \hfill \\d_{j}^{l} {\left\{ {\begin{array}{*{20}l} {d_{j} ,} \hfill & {l = 0} \hfill \\ {\left( {1 - \alpha_{j}^{l} } \right)d_{j - 1}^{l - 1} + \alpha_{j}^{l} d_{j}^{l - 1} ,{ }} \hfill & {l = 1,2, \cdots ,k;j = i - k + l, \cdots ,i} \hfill \\ \end{array} } \right.} \hfill \\ {\alpha_{j}^{l} = \frac{{u - u_{j} }}{{u_{j + k + 1 - l} - u_{j} }}} \hfill \\ \end{array} } \right.$$

Within the mathematical formulation, ${d}_{i}\in {R}^{M\times 1}$ signifies the control vertices governing the joint spline trajectory, while $u\in \left[{u}_{i},{u}_{i+1}\right]$ corresponds to the normalized temporal node vector associated with the ith segment of the spline curve. The term ${N}_{i,k}(u)$ denotes the kth-order normalized B-spline basis function.

Based on the local support property of B-spline curves, the rth derivative of *p(u)* is obtained as:

7 $${p}^{r}\left(u\right)=\sum_{j=i-k+r}^{i}{d}_{j}^{r}{N}_{j,k-r}\left(u\right), {u}_{i}\le u\le {u}_{i+1}$$

where $$d_{j}^{l} = \left\{ {\begin{array}{*{20}l} {d_{j} , } \hfill & { l = 0} \hfill \\ {\left( {k + 1 - l} \right)\frac{{d_{j}^{l - 1} - d_{j - 1}^{l - 1} }}{{u_{j + k + 1 - l} - u_{j} }}, } \hfill & { l = 1,2, \cdots ,r;j = i - k + l, \cdots ,i} \hfill \\ \end{array} } \right.$$

### Multi-objective trajectory optimization model

#### Position constraints

In the robotic bricklaying operation, the brick’s motion trajectory is constrained within the rectangular region delineated by the dashed lines in Fig. 2a. Consequently, the spatial boundary conditions for the X and Z axes of the ith spline segment ${x}_{i}(t)$ and ${z}_{i}(t)$ are defined as:

8 $$\left\{ {\begin{array}{*{20}c} {x_{{{\mathrm{min}}}} \le x_{i} \left( t \right) \le x_{{{\mathrm{max}}}} } \\ {z_{{{\mathrm{min}}}} \le z_{i} \left( t \right) \le z_{{{\mathrm{max}}}} } \\ \end{array} } \right.{ }i = b, \cdots ,d$$

In the mathematical formulation, *b* and *d* correspond to the spline trajectory segments housing the path points B and D respectively.

Furthermore, during the brick lifting phase, it is necessary to ensure that AB follows a straight-line motion, hence:

9 $$\left|{x}_{i}(t)-{x}_{s}\right|\le {\varepsilon}_{\mathrm{m}\mathrm{a}\mathrm{x}} i=a,\cdot \cdot \cdot ,b$$

where ${\varepsilon}_{max}$ denotes the maximum straightness error of the straight-line trajectory segment AB. ${x}_{s}$ is the X-coordinate of the segment AB on the straight line.

#### Velocity, acceleration, and acceleration constraints

Due to mechanical limitations of the wall-building robot, all joint motion parameters are constrained within reasonable ranges during bricklaying. The following constraints must be satisfied:

10 $$\left\{ {\begin{array}{*{20}l} {\dot{q}_{m}^{L} \le \dot{q}_{mi} \left( t \right) \le \dot{q}_{m}^{U} } \hfill \\ {\ddot{q}_{m}^{L} \le \ddot{q}_{mi} \left( t \right) \le \ddot{q}_{m}^{U} } \hfill \\ {\left| {\dddot q_{mi} \left( t \right)} \right| \le J^{MAX} } \hfill \\ \end{array} } \right.\;\;\;\;\;\;\;\;\;\;\;\;i = 0, \cdots ,n;j = 1, \cdots ,m$$

where ${\dot{q}}_{mi}(t)$ denotes the velocity of joint $m,^\circ /s$; $\ddot{q}_{mi}$*(t)* denotes the acceleration of joint *m*, $^\circ /{s}^{2}$; $\dddot q_{mi}$*(t)* denotes the jerk of joint *m*, $^\circ /{s}^{3}$; ${\dot{q}}_{m}^{L}$, ${\dot{q}}_{m}^{U}$, ${\ddot{q}}_{m}^{L}$, ${\ddot{q}}_{m}^{U}$ denote the upper and lower limits for velocity and acceleration of joint *m*; ${J}^{MAX}$ represents the maximum jerk.

#### Torque constraints

The dynamic equations for the wall-building robot are as follows:

11 $$D(q)\ddot{q}+C(q,\dot{q})\dot{q}+g(q)=\tau$$

where *τ* denotes the joint torque; *D(q)* represents the *n× n* mass matrix of the robotic arm; $C(q,\dot{q})$ signifies the *n× 1* vector of centrifugal and Coriolis forces; and *g(q)* indicates the *n× 1* gravitational force vector. Since robot dynamics constraints primarily address torque variations, this paper employs fixed dynamic parameters while neglecting the effects of friction and payload changes.

During bricklaying, to prevent damage to the robotic arm from excessive torque generation, corresponding joint torques must be limited. The torque constraint condition is as follows:

12 $$\left|\tau \right|\le {\tau}_{\mathrm{m}\mathrm{a}\mathrm{x}}$$

where ${\tau}_{max}$ denotes the constraint value for the joint torque.

Unlike traditional single-objective trajectory optimization, this study’s wall-building robot must simultaneously enhance laying efficiency, minimize energy consumption, and achieve the smoothest possible motion trajectory for brick placement. Furthermore, based on the previously constructed Kriging model, it is also necessary to minimize both the laying error Error and the reaction force Fmax. Thus, the trajectory optimization problem in this study constitutes a complex mixed-variable nonlinear issue involving five objective functions, yielding a Pareto frontier with irregular shape. As a result, the FEPSO method is utilized to address the trajectory optimization problem. The constructed multi-objective trajectory optimization model for segments ABCDE is as follows:

13 $${\mathrm{min}}\left\{ {\begin{array}{*{20}l} {{\mathrm{Error}} = f_{1} \left( {t_{1} ,{ }t_{2} ,{ }t_{3} ,x,{ }l,{ }v,{ }h} \right)} \hfill \\ {{\mathrm{Time}} = f_{2} \left( {t_{1} ,{ }t_{2} ,{ }t_{3} ,{ }x,{ }l,{ }v,{ }h} \right) = T_{{{\mathrm{free}}}} { } + { }T_{{{\mathrm{DE}}}} = \mathop \sum \limits_{i = 0}^{n - 1} \left( {t_{i + 1} - t_{i} } \right) + \left( {\Delta x + l} \right)/v} \hfill \\ {{\mathrm{SQ}} = f_{3} \left( {t_{1} ,{ }t_{2} ,{ }t_{3} ,x,{ }l,{ }v,{ }h} \right) = \mathop \sum \limits_{m = 1}^{M} \sqrt {\frac{1}{{T_{{{\mathrm{free}}}} }}\mathop \smallint \limits_{0}^{{T_{{{\mathrm{free}}}} }} (a_{i} )^{2} dt} } \hfill \\ {{\mathrm{Fmax}} = f_{4} \left( {t_{1} ,{ }t_{2} ,{ }t_{3} ,x,{ }l,{ }v,{ }h} \right)} \hfill \\ {{\mathrm{SJ}} = f_{5} \left( {t_{1} ,{ }t_{2} ,{ }t_{3} ,x,{ }l,{ }v,{ }h} \right) = \mathop \sum \limits_{m = 1}^{M} \sqrt {\frac{1}{{T_{{{\mathrm{free}}}} }}\mathop \smallint \limits_{0}^{{T_{{{\mathrm{free}}}} }} (j_{i} )^{2} dt} } \hfill \\ \end{array} } \right.\;\;s.t\left\{ {\begin{array}{*{20}l} {x_{{{\mathrm{min}}}} \le x_{i} \left( t \right) \le x_{{{\mathrm{max}}}} } \hfill \\ {z_{{{\mathrm{min}}}} \le z_{i} \left( t \right) \le z_{{{\mathrm{max}}}} } \hfill \\ {\left| {x_{i} \left( t \right) - x_{s} } \right| \le \varepsilon_{{{\mathrm{max}}}} } \hfill \\ {\dot{q}_{m}^{L} \le \dot{q}_{mi} \left( t \right) \le \dot{q}_{m}^{U} } \hfill \\ {\ddot{q}_{m}^{L} \le \ddot{q}_{mi} \left( t \right) \le \ddot{q}_{m}^{U} } \hfill \\ {\left| {\dddot q_{mi} \left( t \right)} \right| \le J^{MAX} } \hfill \\ {\left| \tau \right| \le \tau_{{{\mathrm{max}}}} } \hfill \\ {252 \le x \le 400} \hfill \\ {0.1 \le l \le 10} \hfill \\ {10 \le h \le 30} \hfill \\ {10 \le v \le 150} \hfill \\ {f_{1} > 0} \hfill \\ {f_{4} < 9500} \hfill \\ \end{array} } \right.$$

In the equation, ${t}_{1}$, ${t}_{2}$, and ${t}_{3}$ represent the motion times of path points B, C, and D, respectively, serving as optimization variables; ${T}_{free}$ represents the movement time of the robot during the free movement phase, and ${T}_{DE}$ represents the movement time of the robot during the DE phase; ${a}_{i}$ and ${j}_{i}$ denote the acceleration and jerk of each joint; *Time* measures the motion duration of the robot’s bricklaying process, evaluating motion efficiency; *SQ* quantifies the mean joint acceleration to evaluate energy efficiency; The primary energy consumption in robotic joints is attributed to motor copper losses, which are proportional to the square of angular acceleration. The root-mean-square acceleration, defined as the time-average of angular acceleration squared, is directly correlated with cumulative energy consumption over a motion cycle while accounting for random fluctuations in instantaneous acceleration, making it an effective metric for evaluating joint energy consumption. *SJ* characterizes the average joint pulsation to assess motion fluidity.

### FEPSO algorithm

Traditional PSO algorithms are prone to premature convergence and getting trapped in local optima when solving high-dimensional nonlinear multi-objective optimization problems; their performance is particularly limited when addressing optimization problems involving irregularly shaped Pareto fronts. To this end, this paper employs an improved particle swarm optimization algorithm (FEPSO) based on fractal Brownian motion, incorporating fractal randomness into the particle search process to balance randomness, self-similarity, and long-term memory, thereby significantly enhancing global optimization capability and convergence stability. Building upon the classical PSO framework, FEPSO introduces a two-stage search mechanism driven by fractal Brownian motion. The algorithm is divided into three stages:

1. Individual fractal self-search: Particles autonomously explore based on a fractal random strategy to enhance population diversity and prevent premature convergence;
2. Optimal individual self-optimization: Refined fractal updates are applied to the global optimal solution to enhance local development accuracy;
3. Adaptive parameter mechanism: The fluctuation coefficient is dynamically adjusted according to the population’s evolutionary state, ensuring that the search step size better aligns with the optimization scenario.

Compared with traditional PSO and MOPSO, the key improvements of FEPSO lie in: overcoming the limitation of fixed random terms by adopting trend-based fractal random updates, making it better suited for high-dimensional nonlinear optimization; a three-stage search structure that balances global exploration with local refinement, delivering superior performance on irregular Pareto front problems; and adaptive fluctuation parameters that dynamically adjust the search step size based on the population’s evolutionary state.

**Fig. 5:**
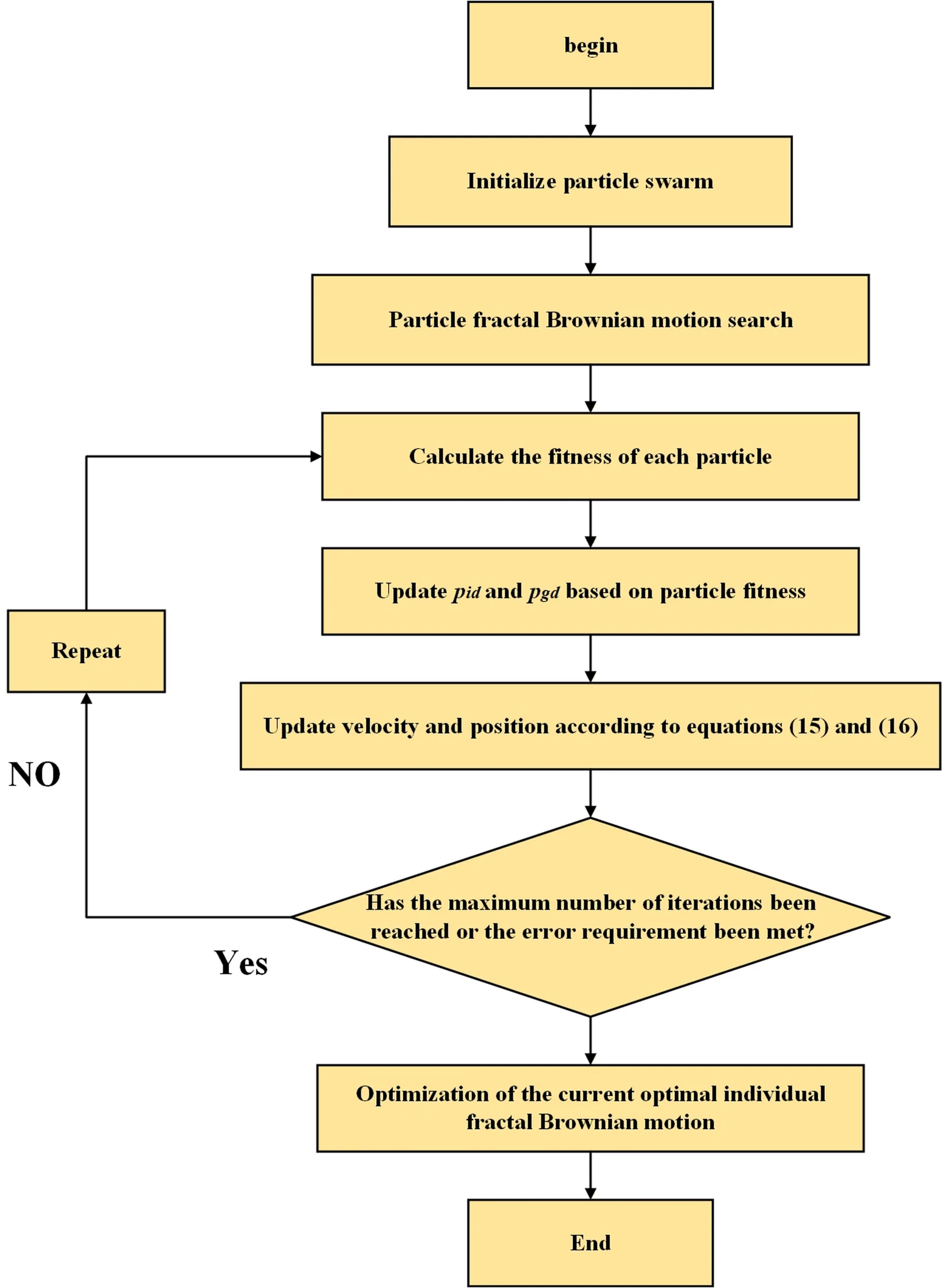
Particle swarm optimization algorithm flowchart using fBm search method.

FEPSO retains the classic PSO’s velocity-position iteration framework, incorporates a fractal stochastic term into velocity updates, and introduces an additional self-optimization mechanism for optimal individuals. The algorithmic flow is illustrated in Fig. 5.

During the initial phase of the fractal evolutionary algorithm, each particle undergoes a self-search process based on fBm. Following this process, the fitness value of each particle is calculated. Subsequently, the individual optimal solution position ${p}_{id}$ and the population optimal solution position ${p}_{gd}$ are updated according to the particles’ fitness values. Then, the velocity and position of the particles are updated. This phase aligns with the traditional particle swarm algorithm. Upon reaching the maximum iteration count or satisfying the required error threshold, the current optimal individual undergoes self-optimization based on fBm. The self-optimization search phase for individual particles, modeled after stock price fluctuation Equation, comprises two components: reflecting the overall trend of the objective function and incorporating random disturbance terms $BH\left(t\right)$. The detailed mathematical derivations of the algorithm, formula derivation processes, and parameter definitions are presented in Appendix A.

### TOPSIS algorithm

TOPSIS, formally termed the "Technique for Order Preference by Similarity to Ideal Solution", constitutes a distance-oriented multi-objective evaluation approach^32^. Proposed by Hwang and Yoon in 1981, it is regarded as one of the optimal multi-objective decision models. It ranks solutions by calculating the distance between their objective function values and an ideal solution, thereby approximating the optimal solution during the search process. This algorithm finds extensive application in multi-objective optimization, machine learning, data mining, and related fields. The present study implements the Technique for Order Preference by Similarity to Ideal Solution to evaluate various parameter combinations within the Pareto-optimal frontier, ultimately identifying the most favorable masonry configuration.

## Results and discussion

### Accuracy analysis of the Kriging model

This study employed Leave-One-Out Cross Validation (LOOCV) to rigorously validate the Kriging proxy model, a gold-standard validation method applicable in small-sample, high-dimensional experimental designs. During validation, only one sample group was retained as an independent test set each time, while the remaining 17 groups were used to train the Kriging model and generate predictions; this process was repeated iteratively until every sample had been utilized once for validation, after which all prediction results were combined to calculate model accuracy metrics. This approach eliminates data waste and random division bias, maximizes the utilization of limited experimental data, and provides an objective assessment of the model’s interpolation accuracy and generalization performance, making it well-suited for the modeling scenario with four-dimensional inputs and 18 experimental samples in this study.

The study employed three statistical metrics—root mean square error (RMSE), mean absolute error (MAE), and mean absolute percentage error (MAPE)—to quantitatively evaluate prediction accuracy^33,34^. Detailed accuracy metrics are presented in Table 4. For the response variable Fmax, the model’s RMSE was 78.3621, MAE was 56.2470, and MAPE was merely 0.7218%; for the response variable Error, the model’s RMSE was 0.2156, MAE was 0.1587, and MAPE was 18.62%. All error metrics were at extremely low levels, demonstrating that the Kriging model can accurately fit multivariate coupled response patterns with excellent modeling performance.

**Table 4:** Prediction accuracy evaluation results.

Response	RMSE	MAE	MAPE (%)	Mean prediction variance	Average half-width of 95% confidence interval
Fmax	78.3621	56.2470	0.7218	0.004276	0.1283
Error	0.2156	0.1587	18.6200	0.005189	0.1415

We further conducted a sensitivity analysis of model accuracy with respect to sample size^35^. The Kriging model is a high-precision interpolation model suitable for small sample sizes, exhibiting significantly lower sensitivity to sample size compared to data-driven models such as neural networks and random forests; when the sample size falls below 10–12 groups, correlation in the four-dimensional input space becomes insufficiently captured, leading to rapid increase in model error; this study employed 18 experimental samples uniformly covering the design space, which adequately characterized both main effects and interaction effects of variables, resulting in model accuracy reaching a saturated level of stability; sensitivity tests demonstrated that adjusting the sample size within the current range did not significantly affect model error, confirming that a sample size of 18 groups is sufficient, appropriate, and free from overfitting risks.

The Kriging model inherently possesses the capability to quantify uncertainty, enabling direct calculation of the 95% confidence interval via mean squared error (MSE), thereby facilitating visualization and quantification of uncertainty^36^. Near sample points: the prediction variance approaches zero, with an extremely narrow confidence interval indicating minimal uncertainty; within the design space: the prediction variance is low, the confidence interval is compact, and prediction reliability is high; the mean prediction variance for Fmax is 0.004276, with a mean half-width of the 95% confidence interval of 0.1283; the mean prediction variance for Error is 0.005189, with a mean half-width of the 95% confidence interval of 0.1415. The globally interpolated confidence intervals are compact and exhibit low prediction variance, demonstrating low model uncertainty and highly reliable prediction results.

After completing the Kriging surrogate model, its accuracy must meet requirements before proceeding with subsequent optimization analyses. The model’s accuracy is evaluated using data points, employing the cross-correlation coefficient ${R}^{2}$ and establishing the model evaluation Eq. (14) to assess the surrogate model’s precision. The metric ${R}^{2}$ quantifies the consistency between projected and measured values, illustrating the Kriging model’s adherence to experimental results.

14 $${\mathrm{R}}^{2}=1-\frac{{\sum}_{i=1}^{M}{\left({g}_{i}-{\widehat{g}}_{i}\right)}^{2}}{{\sum}_{i=1}^{M}{\left({g}_{i}-{\overline{g} }_{i}\right)}^{2}}$$

where *M* stands for the quantity of test specimens utilized in accuracy assessment; ${g}_{i}$ embodies the genuine output of the ith verification case; ${\widehat{g}}_{i}$ refers to the Kriging approximation for that instance; whereas ${\overline{g} }_{i}$ computes the mean of all experimental observations. The ${R}^{2}$ metric operates within 0–1 bounds, with increasing values denoting enhanced reliability.

**Fig. 6:**
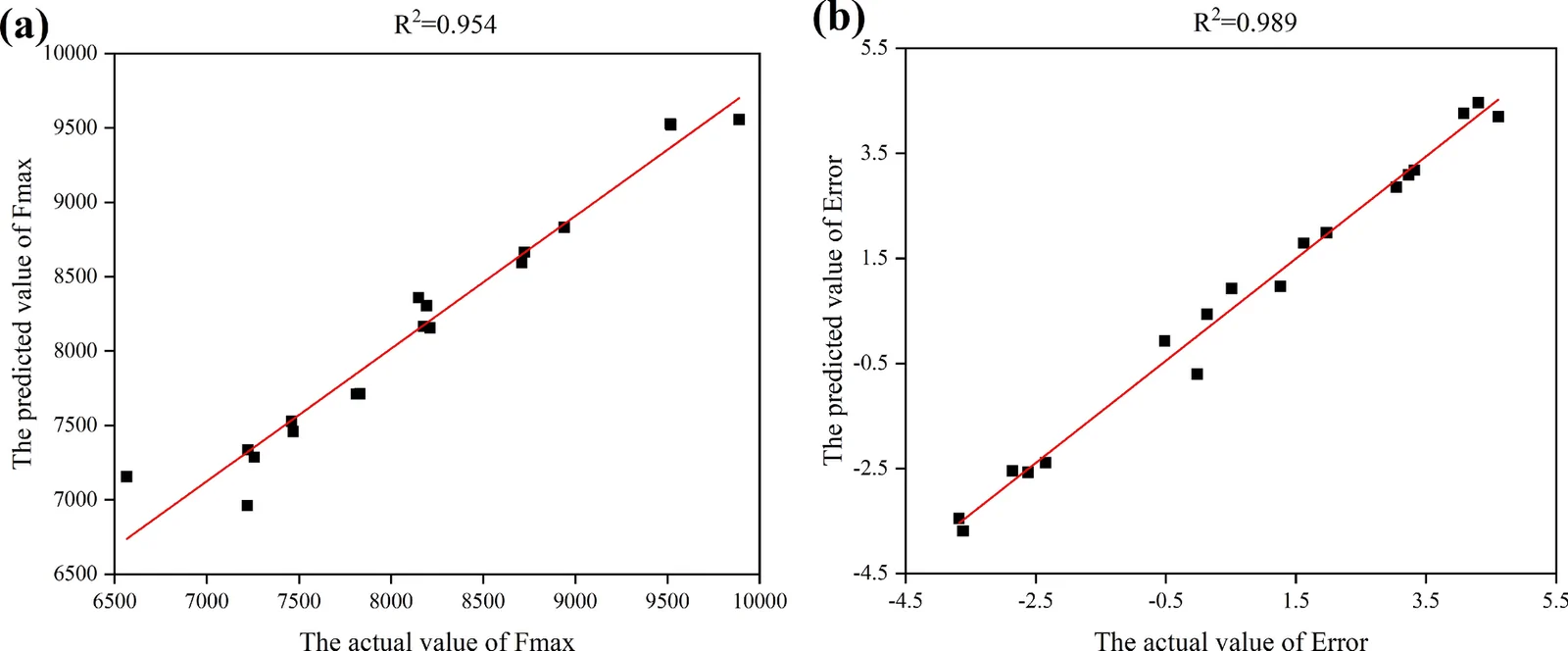
Kriging model correlation coefficients. (**a**) Fmax. (**b**) Error.

**Fig. 7:**
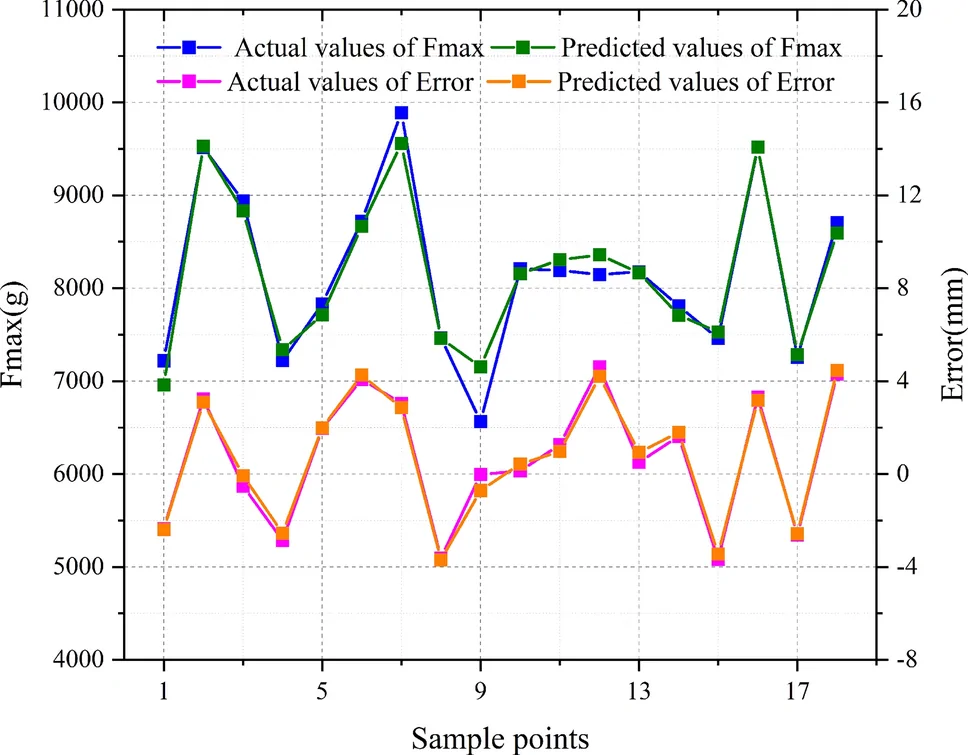
Performance evaluation of the Kriging model.

The validation results of the Kriging model are shown in Fig. 6. Calculated using Eq. (14), the evaluation coefficients ${R}^{2}$ for Fmax and Error are 0.954 and 0.989, respectively. This indicates that the Kriging model exhibits high fitting accuracy and meets analytical requirements. Furthermore, the points in the figure are observed to lie substantially along a straight line, demonstrating that the Kriging model’s predicted values align well with experimental data. Figure 7 displays the experimental data used to validate the Kriging model’s accuracy alongside the model’s predicted values. It is apparent that the computed results for both performance metrics correspond well with actual experimental values, with only a few scattered points. This demonstrates that, provided sufficient training data, the Kriging surrogate model achieves high accuracy in predicting linear or nonlinear, direct or indirect relationships among multiple parameters. The results confirm that the established Kriging model is suitable for practical masonry construction applications, with reliability meeting the requirements for implementation.

### Influence of design variables on performance

**Fig. 8:**
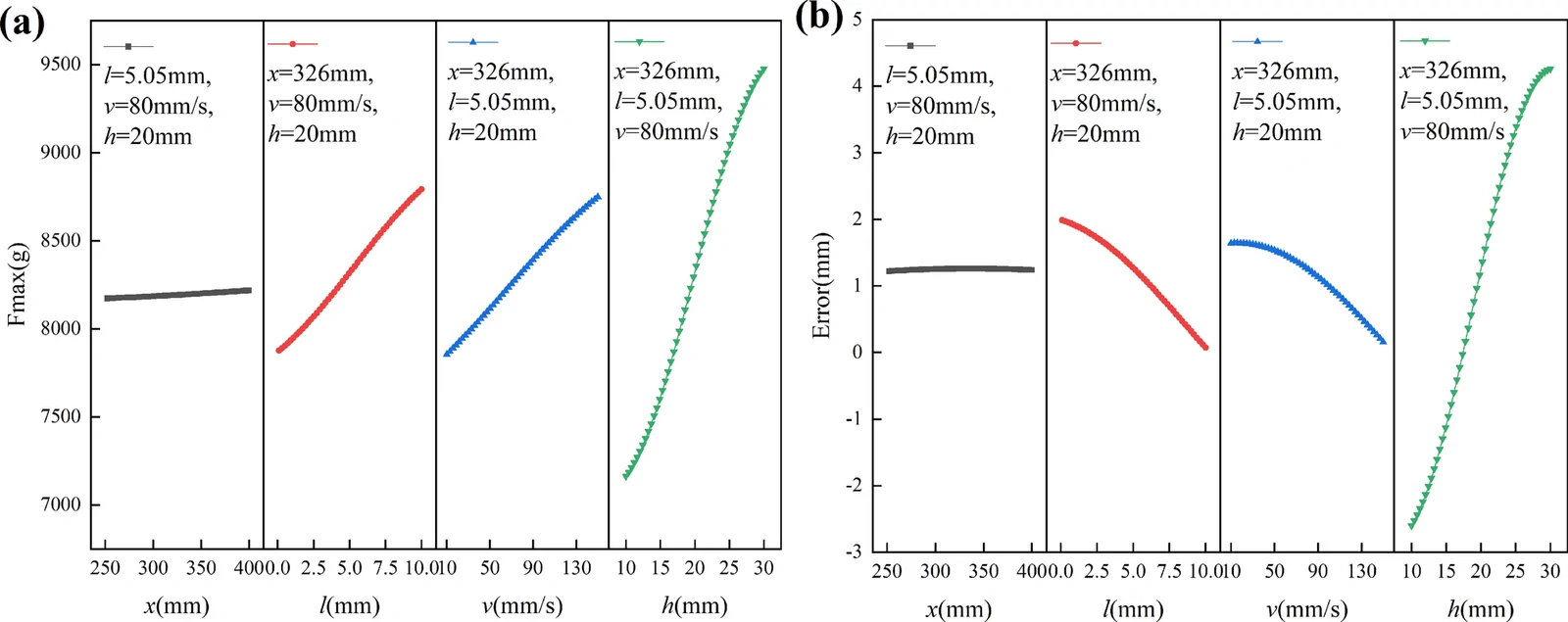
Influence of single design variables on objective functions. (**a**) Fmax. (**b**) Error.

We utilized the controlled variable approach to examine the influence of each design parameter on the performance metric, producing the variation trends depicted in Fig. 8. The results clearly demonstrate that augmenting the mortar layer thickness results in higher maximum contact forces while concurrently amplifying masonry inaccuracies. This effect is pronounced because thicker cement mortar layers result in more severe compression of the mortar, thereby exerting greater contact forces on the robotic arm. Concurrently, increased viscoelastic damping on the robotic arm makes it more difficult for bricks to reach their target positions, leading to larger masonry errors. The position compensation variable and laying speed exhibit a linear positive correlation with Fmax and a negative correlation with Error, with their effects also being pronounced. The phenomenon occurs as elevated positional compensation subjects the cement mortar to more pronounced compression, thereby generating heightened contact forces on the robotic arm. This intensified compression causes the mortar to be more firmly pressed by the bricks, thereby minimizing placement inaccuracies. Under consistent conditions, faster robotic arm movement leads to an augmented mortar compression rate. For viscoelastic materials like cement mortar, longer exposure time enhances stress relaxation effects. Consequently, shorter compression duration manifests as greater contact force. The mortar is compressed more tightly by the bricks, resulting in smaller masonry errors. With increasing masonry distance, both Fmax and Error show minimal variation, suggesting masonry distance exerts negligible influence on these parameters. Hence, to minimize maximum contact forces between the robot and the environment while enhancing masonry accuracy, it is crucial to optimize the interplay among these three variables.

To gain a clearer and more intuitive understanding of how the position compensation variable, laying speed, and mortar application thickness affect Fmax and Error, response surface plots were generated between two design variables and the objective responses. The third variable and laying distance were fixed at their range averages. For each response surface plot, contour plots were also generated to identify the optimal operating region within the given parameter ranges.

**Fig. 9:**
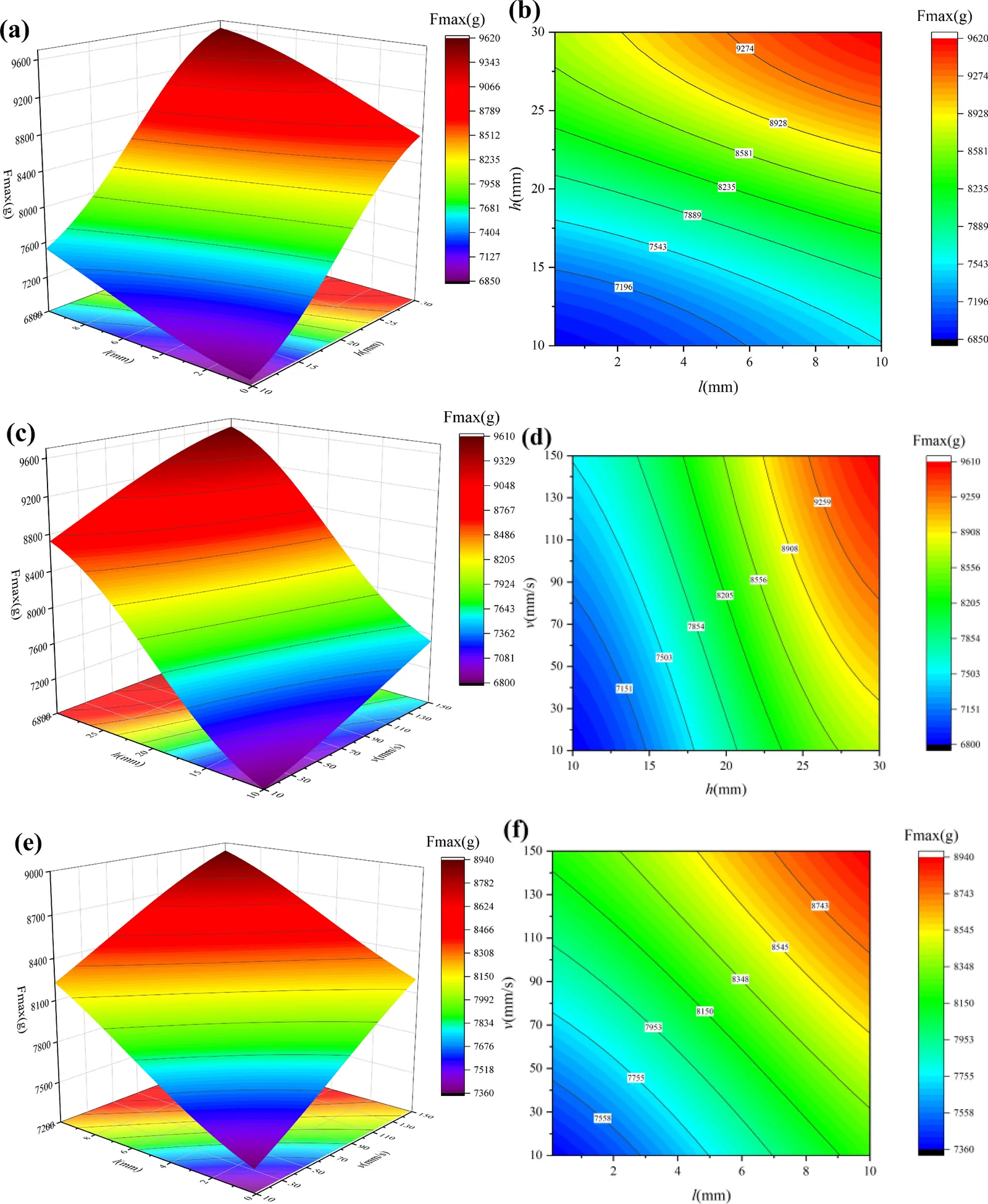
3D response surface plots and 2D contour plots of maximum contact force under independent variable interactions. (**a**) Response surface plot: *l* versus *h*. (**b**) Contour plot: *l* versus *h*. (**c**) Response surface plot: *h* versus *v*. (**d**) Contour plot: *h* versus *v*. (**e**) Response surface plot: *l* versus *v*. (**f**) Contour plot: *l* versus *v*.

The visualization in Fig. 9 provides both three-dimensional response surfaces and two-dimensional contour representations for maximum contact force, analyzing position compensation, masonry velocity, and mortar thickness. Figure 9a clearly indicates that higher values in both position compensation and mortar thickness contribute to increased contact force, showing distinct interdependency. Their contour curvature also indicates these parameters significantly influence Fmax. Figure 9b shows that due to the pronounced curvature of both the position compensation variable and mortar application thickness, the maximum value is achieved within the existing boundaries. For maximum contact force, the contour plot indicates that the effect of mortar application thickness is greater than that of the position compensation variable. Similarly, increases in both masonry speed and mortar application thickness significantly influence maximum contact force. Both factors increase Fmax, exhibiting a clear interaction, with the maximum Fmax occurring within the existing boundaries (Fig. 9c). For maximum contact force, the contour plot indicates that the effect of mortar spread thickness is greater than that of laying speed (Fig. 9d). Figure 9e shows that increases in both the position compensation variable and laying speed also lead to higher maximum contact force, though their interaction is not pronounced. The contour plot in Fig. 9f indicates that the effect of the position compensation variable on maximum contact force is roughly equivalent to that of laying speed.

**Fig. 10:**
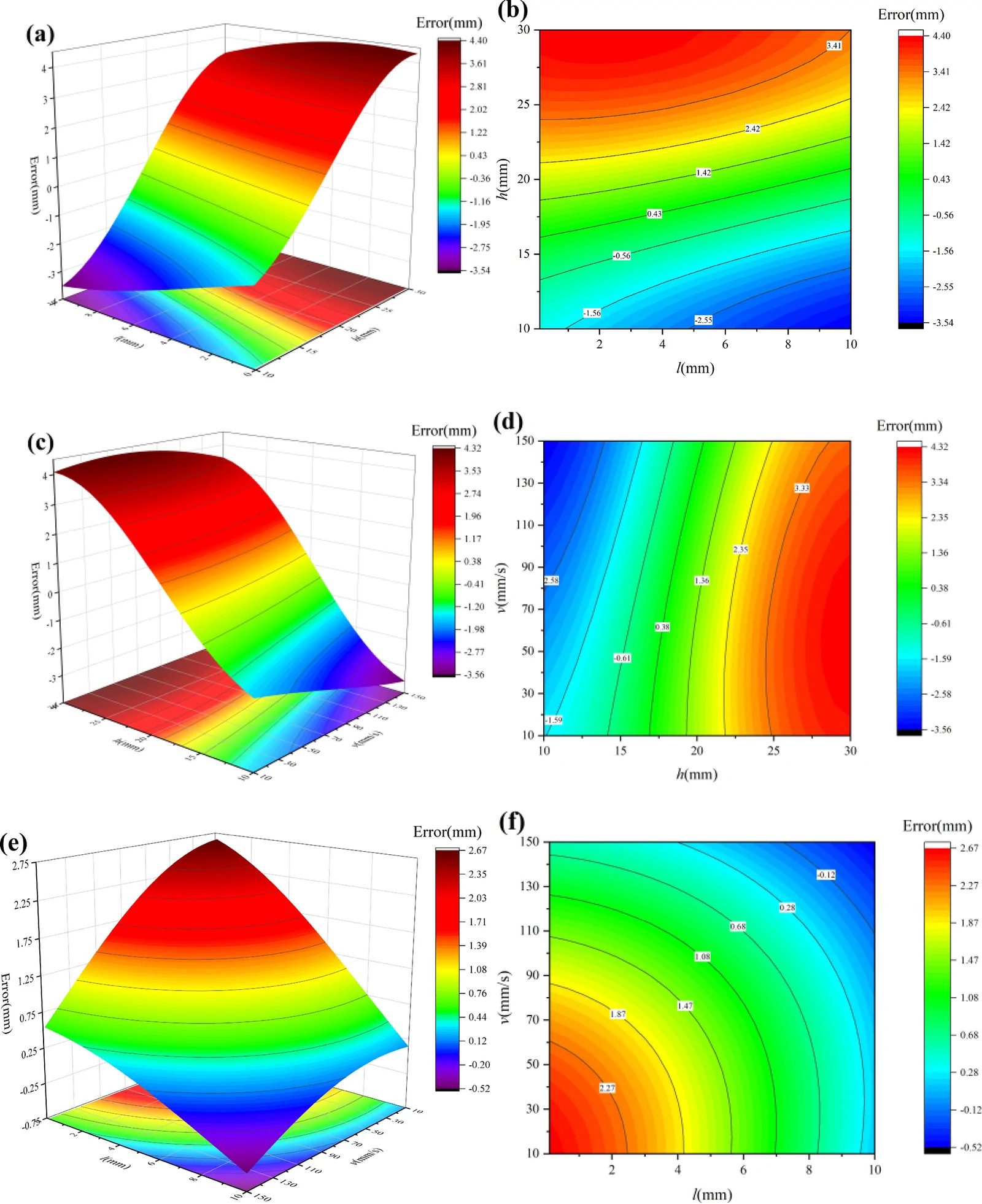
3D response surface and 2D contour plots of masonry errors under variable interactions. (**a**) Response surface plot: *l* versus *h*. (**b**) Contour plot: *l* versus *h*. (**c**) Response surface plot: *h* versus *v*. (**d**) Contour plot: *h* versus *v*. (**e**) Response surface plot: *l* versus *v*. (**f**) Contour plot: *l* versus *v*.

Figure 10 illustrates the three-dimensional response surfaces and corresponding two-dimensional contour mappings for masonry deviation across three key factors: position compensation level, masonry velocity, and mortar thickness distribution. Figure 10a reveals that increasing mortar application thickness and decreasing position compensation variable both elevate masonry error, indicating a clear interaction between them. The pronounced curvature of their contour lines further confirms the significant influence of these parameters on Error. Figure 10b indicates that due to the significant curvature of both the position compensation variable and mortar application thickness, the maximum values are obtained within the existing boundaries. For masonry error, the contour plot shows that the effect of mortar application thickness is greater than that of the position compensation variable. Correspondingly, both placement velocity and mortar layer thickness demonstrate significant effects on laying error. Higher mortar thickness and lower laying speed result in greater laying error. A clear interaction exists between them, with maximum error occurring within the existing boundaries (Fig. 10c). For laying error, the contour plot indicates that the effect of mortar thickness is greater than that of laying speed (Fig. 10d). The contour lines within the contour plot represent the forecasted Error values throughout different areas of the domain. Figure 10e indicates that increasing the position compensation variable and masonry speed consistently reduces masonry error, though their interaction is not pronounced. The contour lines in Fig. 10f show that the impact of the positional compensation parameter on masonry error is roughly equivalent to that of masonry speed.

### Comparative analysis of various algorithms

To assess the effectiveness of FEPSO in solving complex multi-objective trajectory optimization scenarios, this study selected the most representative NSGA-II and MOPSO as comparison algorithms based on current major research directions. Multi-objective optimization calculations were performed for each algorithm. The corresponding parameter settings are shown in Table 5. Other specific parameters of the FEPSO algorithm are detailed in its algorithm introduction section.

**Table 5:** Parameter settings for each algorithm.

Parameter settings	NSGA-II	Parameter settings	MOPSO	FEPSO
Population size	100	Population Size	100	100
Number of iterations	100	Number of Iterations	100	100
Mutation probability	0.02	Inertia Weight	0.8	0.8
Crossover probability	0.8	Learning Factors ${c}_{1}$,${c}_{2}$	2	2

We implement four evaluation benchmarks in this research: the convergence metric (CM)^37^, the diversity metric (DM)^38^, the spacing metric (SM)^39^, and the inverse generational distance (IGD)^40^ to compare algorithm efficiency. CM, DM, and SM are singular metrics, in contrast to IGD which is a multifaceted metric. A minimized CM points to optimal convergence of the Pareto front. An amplified DM highlights broader diversity within the Pareto front. A lessened SM demonstrates improved spacing in the Pareto front. A curtailed IGD value evidences superior global performance. Each algorithm underwent ten iterations, with averages computed to maintain consistency. The recorded execution data appears in Table 6.

**Table 6:** Evaluation metric values for each algorithm.

Algorithms	Evaluation Indicators			
	*CM*	*DM*	*SM*	*IGD*
FEPSO	0.0549	0.5104	1.0313	2.7563
NSGAII	0.5273	0.1686	2.3842	6.5545
MOPSO	0.6536	0.1173	2.7473	7.2253

**Fig. 11:**
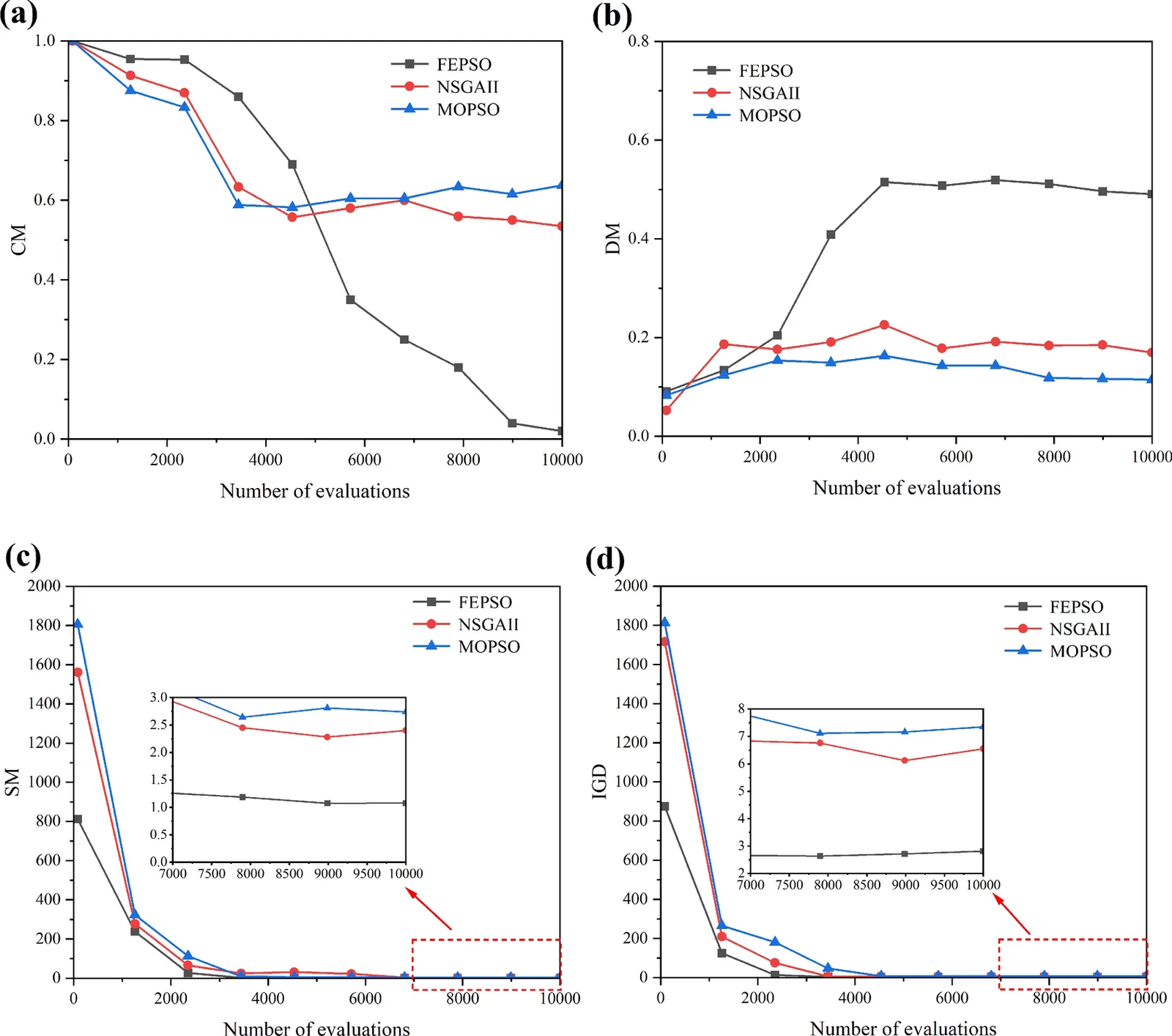
Trends in evaluation metrics for different algorithms. (**a**) CM. (**b**) DM. (**c**) SM. (**d**) IGD.

To more intuitively reflect the performance of each algorithm, Fig. 11 presents the trend variations of different evaluation metrics for each algorithm.

**Fig. 12:**
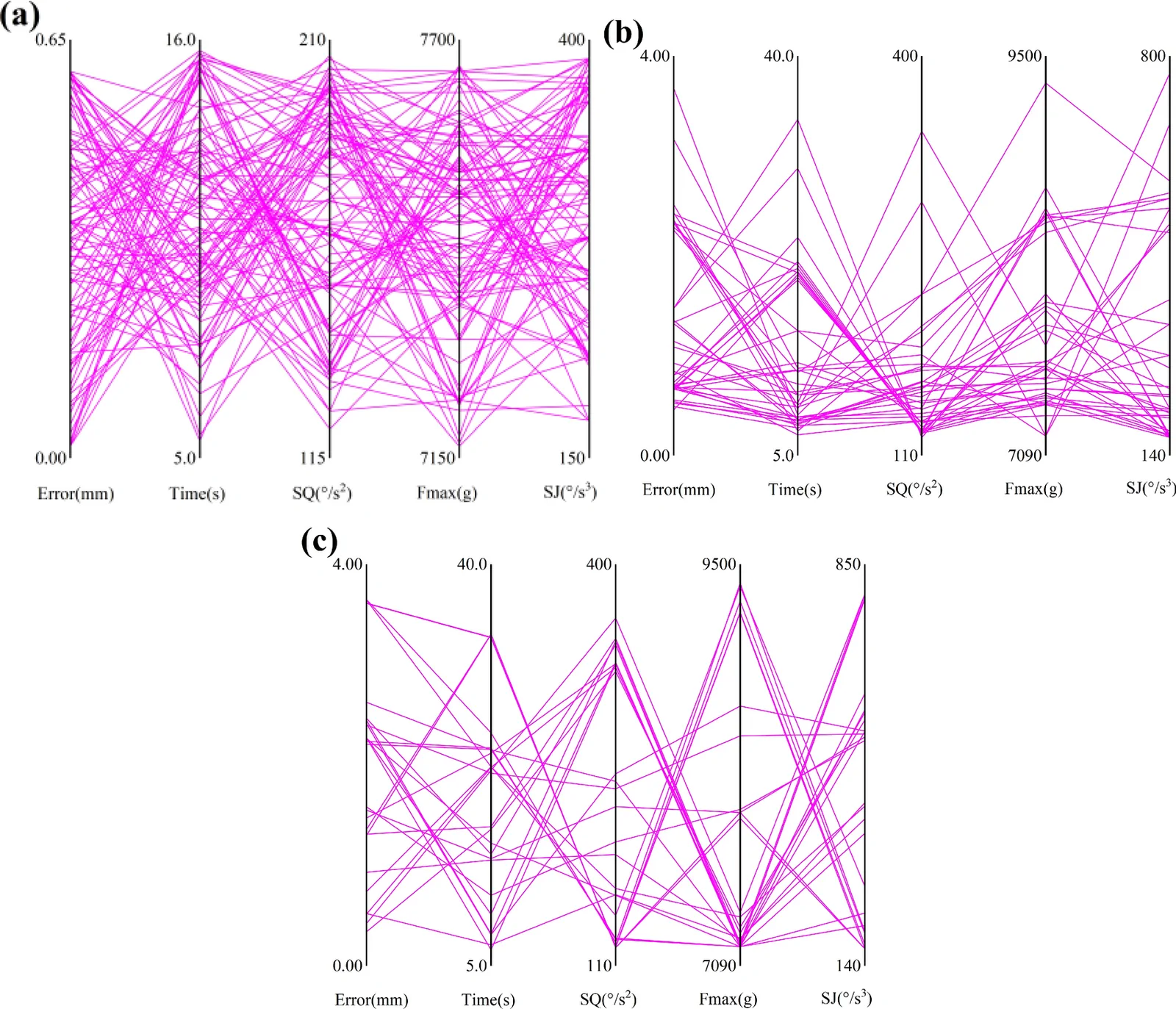
Solution sets in parallel coordinate systems for different algorithms. (**a**) FEPSO. (**b**) NSGA-II. (**c**) MOPSO.

The computational results reveal that the FEPSO algorithm achieves CM, DM, SM, and IGD values of 0.0549, 0.5104, 1.0313, and 2.7563, respectively. These metrics outperform those of the NSGA-II and MOPSO algorithms, demonstrating that the FEPSO algorithm achieves optimal overall performance under the masonry conditions defined by the five optimization objectives in this study. Figure 11 also demonstrates that the FEPSO algorithm achieves the best convergence in the obtained Pareto solution set, as its CM value ultimately approaches zero, whereas the CM values for NSGA-II and MOPSO remain relatively high. Similarly, the FEPSO algorithm yields the most balanced and diverse distribution of the Pareto solution set. To provide a more intuitive understanding of each algorithm’s performance, the solution sets obtained by each algorithm are presented in Fig. 12. When dealing with multi-objective optimization tasks having three or more objectives, the Pareto solution set is graphed as a parallel coordinate visualization with multiple dots. Every dot encapsulates a separate solution variant^41^.

The solution set generated by the FEPSO algorithm demonstrates an approximately interconnected arrangement. The Pareto solution set displays satisfactory diversity, with evenly distributed solutions throughout the set. Furthermore, the values computed by the algorithm converge within a reasonable range, further demonstrating the distinct advantage of the FEPSO algorithm in solving optimization problems involving Pareto solution sets with irregular shapes. In the context of the trajectory optimization problem under masonry conditions with five optimization objectives studied in this paper, the objective function exhibits strong nonlinear and non-convex characteristics, with a large number of local optima present in the search space; conventional particle swarm optimization algorithms are prone to premature convergence. Fractal Brownian motion possesses a non-Markov long-memory property, which enhances the exploration capability of particle search compared to Gaussian random perturbations and helps the algorithm escape local optima. Furthermore, the multi-objective trajectory optimization Pareto front lacks regularity and fixed convexity, requiring extremely uniform distribution of solution sets. The irregular continuous perturbation nature of fractal Brownian motion aligns well with the search requirements of an irregular frontier, improving the completeness of solution set coverage. Additionally, this trajectory optimization involves temporal correlations, imposing continuity constraints between consecutive path nodes; the self-similarity property of fractal Brownian motion matches the temporal variation patterns of trajectories, enabling particle updates to better reflect the logic of continuous path evolution and enhancing the efficiency of feasible solution generation. The NSGA-II algorithm features a mature architecture, high code openness, and low computational overhead, making it highly practical for lightweight multi-objective optimization scenarios characterized by low-dimensional constraints, stringent real-time requirements, and limited computing resources. The MOPSO algorithm demonstrates exceptional global search capability; for single-peaked, low-complexity continuous multi-objective problems, it achieves faster convergence with a simple structure that is easy to debug, making it suitable for rapid optimization tasks under straightforward conditions. However, in the masonry application scenario involving five optimization objectives described herein, the Pareto solution sets obtained using both NSGA-II and MOPSO algorithms failed to converge within a reasonable range, rendering it impossible to obtain an optimal solution for the trajectory optimization problem.

### Evaluation of trajectory planning outcomes

Using the Nova5 robot as the experimental subject, the suggested trajectory optimization approach was employed to plan the movement path for a wall-building robot. The path must pass through points A, B, and C to reach the stop position smoothly, with point D being the floating point requiring optimization. The joint angles and constraints at each path point are shown in Table 7. The FEPSO algorithm was applied to optimize the ABCDE segment model, with the resulting Pareto optimal solution set illustrated in Fig. 12a. The visualization reveals inherent trade-offs between the five objective functions, demonstrating that simultaneous minimization of all objectives is unattainable. To address these conflicting relationships, the TOPSIS method was implemented to identify a balanced solution from the Pareto front that aligns with practical masonry specifications. The selected compromise solution’s objective function values and associated design parameters are detailed in Table 8, while the optimal joint velocities at point D are presented in Table 7.

**Table 7:** Joint angles and constraints at path points.

Waypoints	Joint 1	Joint 2	Joint 3	Joint 4	Joint 5	Joint 6
$A/\left(^\circ \right)$	180	− 30.10	− 155.20	95.33	90.00	0
$B/\left(^\circ \right)$	180	7.39	− 145.13	47.77	90.00	0
$C/\left(^\circ \right)$	180	− 11.48	− 79.78	1.33	90.00	0
$D/\left(^\circ \right)$	180	− 61.49	− 30.63	15.68	90.00	0
Speed $v/\left(^\circ /s\right)$	0	− 1.64	− 1.56	− 0.03	0	0
Constraints						
$\left|v\right|/\left(^\circ /s\right)$	–	100	100	100	–	–
$\left|a\right|/\left(^\circ /{s}^{2}\right)$	–	150	150	100	–	–
$\left|j\right|/\left(^\circ /{s}^{3}\right)$	–	1000	1000	500	–	–
$\left|\tau \right|/\left(Nm\right)$	–	100	100	50	–	–

**Table 8:** Objective function and design variables for the compromise solution.

Design variables							Objective functions				
${t}_{1}\left(s\right)$	${t}_{2}\left(s\right)$	${t}_{3}\left(s\right)$	$x\left(mm\right)$	$l\left(mm\right)$	$v\left(mm/s\right)$	$h\left(mm\right)$	$Error\left(mm\right)$	$Time\left(s\right)$	$SQ\left(^\circ /{s}^{2}\right)$	$Fmax\left(g\right)$	$SJ\left(^\circ /{s}^{3}\right)$
1.83	4.12	6.01	341.34	2.35	30.63	13.83	0.08	9.71	130.47	7349.78	213.54

**Fig. 13:**
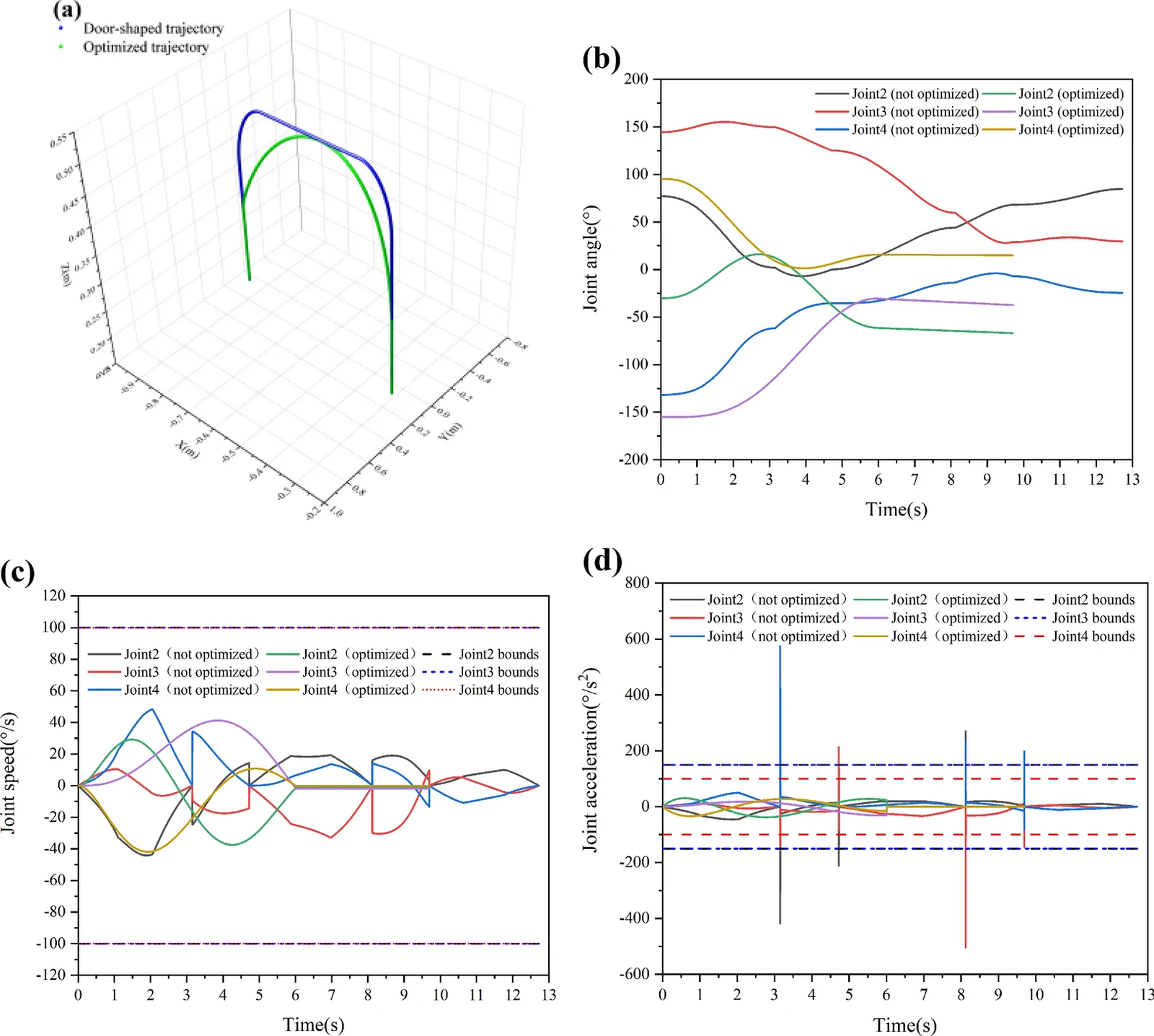
Comparison of motion parameters before and after optimization. (**a**) motion trajectory. (**b**) joint angles. (**c**) joint velocities. (**d**) joint accelerations.

To validate the performance of the suggested trajectory optimization approach, a comparative study was performed between the derived compromise solution and the traditional standard door-shaped trajectory planning technique. The findings are presented in Fig. 13.

It can be observed that under the conventional standard door-shaped trajectory planning method, the robot’s joint spatial trajectory is non-smooth, with discontinuities in joint angular velocity and acceleration. Furthermore, oscillatory phenomena occur in joint acceleration, exceeding the joint constraint values. This readily induces robot vibration, failing to adequately protect components such as motors and reducers. Conversely, the robot’s joint spatial trajectory, angular velocity, and acceleration profiles derived from the 7th-order B-spline curve trajectory planning technique exhibit remarkable smoothness, fully meeting joint limitation requirements. This unequivocally highlights the advantage of the developed motion optimization approach.

**Fig. 14:**
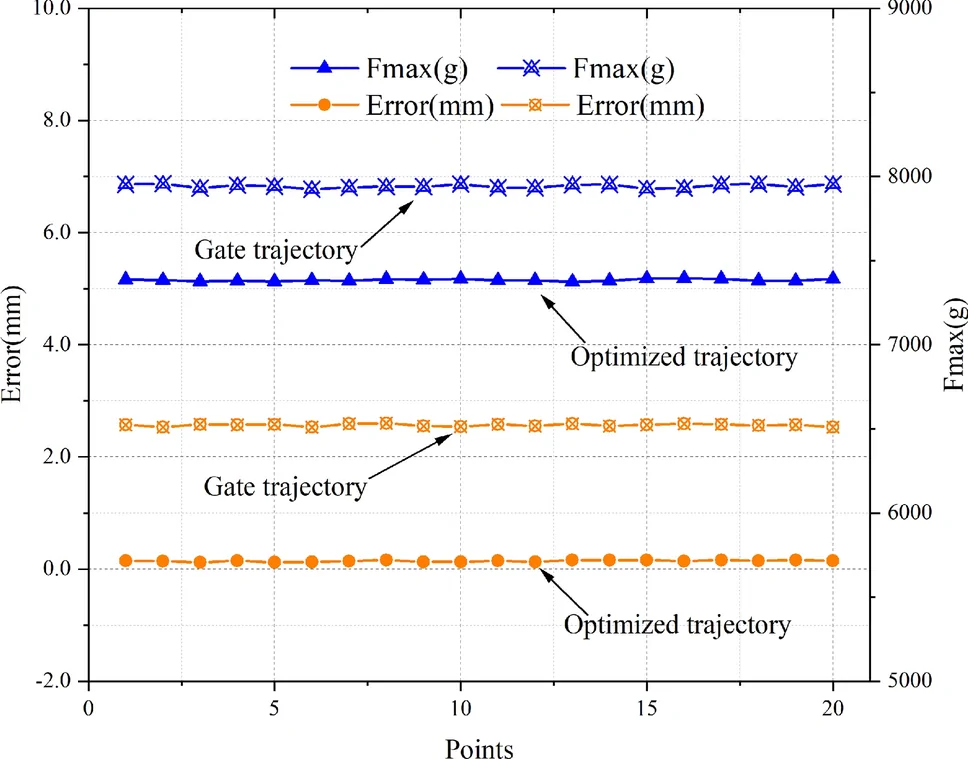
Results of 20 bricklaying attempts before and after optimization.

**Fig. 15:**
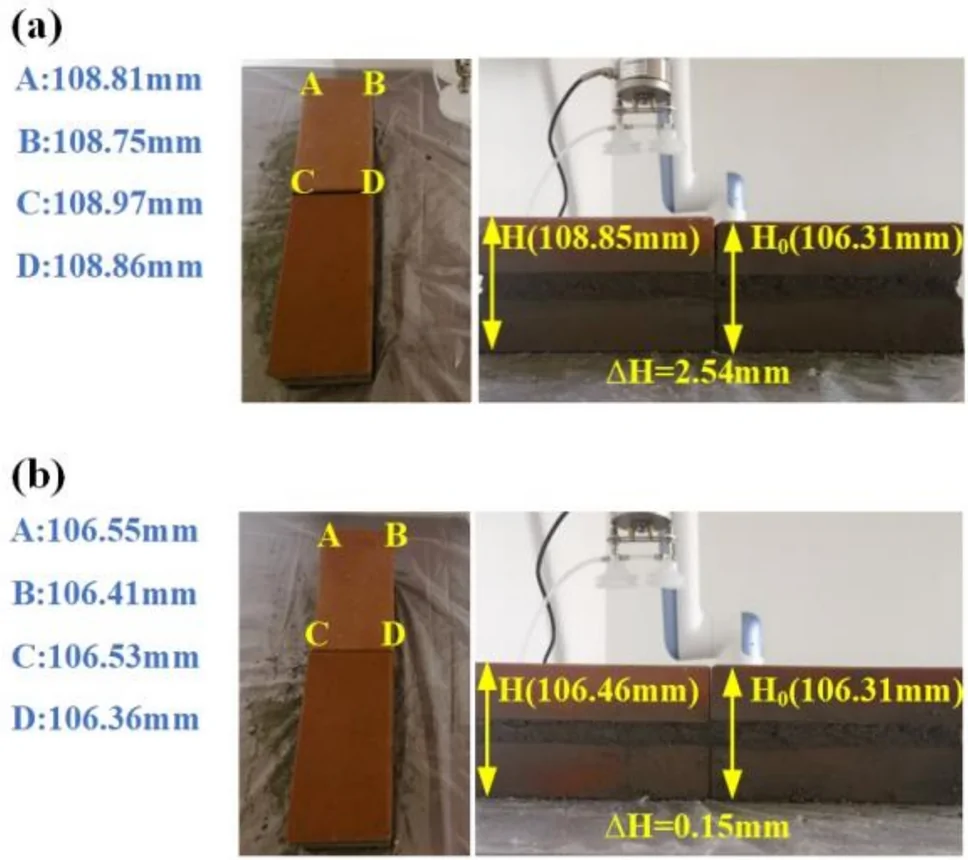
Comparison of bricklaying outcomes pre-optimization and post-optimization. (**a**) pre-optimization. (**b**) post-optimization.

To reduce the randomness of results, the optimized trajectory and the gate-type trajectory were executed repeatedly by the robotic arm 20 times. The experimental results are shown in Fig. 14. This study conducted descriptive statistical analysis of the measurement data: the *Error* for the optimized trajectory was 0.1445 ± 0.0139 (95% confidence interval: 0.1380–0.1510); the *Error* for the gate-shaped trajectory was 2.5655 ± 0.0219 (95% confidence interval: 2.5553–2.5757). The Fmax values for the optimized trajectory were 7384.37 ± 6.03 (95% confidence interval: 7381.55–7387.19), while those for the gate-shaped trajectory were 7943.19 ± 10.45 (95% confidence interval: 7938.30–7948.08). The data exhibited low dispersion and good experimental reproducibility. To determine whether the observed changes were genuine effects, a t-test was performed for statistical significance, yielding P-values of < 0.001 for both Error and Fmax, indicating highly significant differences between the two groups and confirming that the observed numerical variations were not due to random errors. The average values of the maximum contact force and masonry error from these 20 runs are shown in Table 9. Table 9 demonstrates that compared to the door-shaped masonry trajectory, the optimized solution achieved a 23.66% reduction in time, a 29.33% decrease in energy consumption, a 90.47% reduction in smoothness, a 7.03% decrease in maximum contact reaction force with the environment, and a reduction in masonry error from 2.57 to 0.14 mm. This indicates substantial enhancements across all key performance indicators, fulfilling the optimization goals and confirming the efficacy of the developed motion planning technique. Figure 15 displays the actual bricklaying results from one of the experimental trials.

**Table 9:** Performance comparison before and after optimization.

Type	$Error\left(mm\right)$	$Time\left(s\right)$	$SQ\left(^\circ /{s}^{2}\right)$	$Fmax\left(g\right)$	$SJ\left(^\circ /{s}^{3}\right)$
Gate-type trajectory	2.57	12.72	184.63	7942.57	2241.86
Optimized trajectory	0.14	9.71	130.47	7384.37	213.54
Improvement (%)	94.55	23.66	29.33	7.03	90.47

## Conclusion

To improve the wall construction precision of wall-building robots operating in the variable viscoelastic contact conditions of cement mortar, a multi-objective trajectory optimization approach combining Kriging surrogate modeling and fractal evolutionary particle swarm optimization is introduced. A predictive Kriging model for cement mortar behavior was developed, parameter influence analysis was conducted, and the robotic masonry trajectory was systematically refined. Both numerical simulations and physical experiments confirm the validity of the proposed motion planning methodology. Key conclusions are summarized as follows:

1. Orthogonal experimental design and Kriging methods were employed for surrogate modeling of cement mortar, yielding complex correlation coefficients ${R}^{2}$ of 0.954 for Fmax and 0.989 for Error, meeting practical engineering application requirements.
2. The influence of design variables on masonry performance within the Kriging model was analyzed. For both Fmax and Error, the design variable *h* was the most significant factor. As *h* increased, both Fmax and Error gradually rose. As *l* and *v* continued to rise, the maximum force exhibited a steady upward trend, whereas the masonry error demonstrated a consistent downward pattern.
3. Comparative analysis of various algorithms’ performance in the trajectory optimization model was conducted. The FEPSO algorithm demonstrated significant superiority over NSGA-II and MOPSO in solving optimization problems with irregularly shaped Pareto solution sets.
4. The proposed trajectory optimization approach demonstrated superior performance over conventional door-shaped trajectory planning, achieving a 23.66% improvement in masonry efficiency, 29.33% reduction in energy usage, 7.03% decrease in environmental contact forces, and 90.47% enhancement in trajectory smoothness. Additionally, brick placement accuracy significantly improved, with errors dropping from 2.57 to just 0.14 mm.

This pilot investigation addresses brick masonry trajectory planning in nonlinear viscoelastic environments. The FEPSO trajectory optimization framework presented in this paper exhibits universal applicability, with its core algorithm logic not being constrained by specific robot configurations or masonry parameters. For various operating conditions—including different types of masonry robots, specialized block shapes, and varying joint thicknesses—adaptation can be achieved simply by fine-tuning motion constraints and geometric parameters, demonstrating strong cross-platform compatibility and multi-condition adaptability. Addressing practical challenges at construction sites such as temperature/humidity fluctuations, changes in mortar properties, vibrations, and positioning interference, the optimized trajectory balances motion smoothness with positioning accuracy to effectively mitigate common site disturbances. It demonstrates excellent suitability for conventional wall masonry applications, while performance can be further enhanced under extreme conditions by incorporating an online compensation module. The subsequent work will involve constructing a full-scale masonry robot, validating the effectiveness of this method in practical applications, and overcoming key bottlenecks in robotic technology.

## Data Availability

Data will be made available on request.

## Appendix A: Detailed mathematical derivation of the FEPSO algorithm

To clearly elucidate the implementation mechanism of fractal Brownian motion in particle swarm optimization, this appendix presents the complete derivation process of FEPSO, along with parameter definitions and iteration formulas.

## Traditional PSO speed-position update

The traditional particle swarm optimization algorithm performs velocity and position updates using Eqs. 15 and 16:

15 $${v}_{id}^{k+1}=\omega {v}_{id}^{k}+{c}_{1}{r}_{1}\left({p}_{id}-{s}_{id}^{k}\right)+{c}_{2}{r}_{2}\left({p}_{gd}-{s}_{id}^{k}\right)$$

16 $${s}_{id}^{k+1}={s}_{id}^{k}+{v}_{id}^{k+1}$$

In the formula:

*k* is the number of iterations; ${r}_{1},{r}_{2}\sim U\left[\mathrm{0,1}\right]$ are random numbers;

${c}_{1}$ and ${c}_{2}$ are learning factors; *ω* is the inertia weight;

${p}_{id}$ denotes the optimal position for an individual, while ${p}_{gd}$ represents the global optimal position.

To suppress premature convergence, introduce a stochastic update term into the velocity model:

17 $${v}_{id}^{k+1}=\omega {v}_{id}^{k}+{c}_{1}{r}_{1}\left({p}_{id}-{s}_{id}^{k}\right)+{c}_{2}{r}_{2}\left({p}_{gd}-{s}_{id}^{k}\right)+{c}_{3}{r}_{3}$$

where ${c}_{3}$ is a random factor, and ${r}_{3}$∈(− 0.5,0.5).

## Search model driven by fractal Brownian motion

Drawing on the self-similarity and long memory properties of fractal Brownian motion, the particle evolution is modeled as follows:

18 $$\frac{ds\left(t\right)}{s\left(t\right)}=Ldt+\sigma dBH\left(t\right), s\left(0\right)={s}_{0}>0$$

Its analytical solution is:

19 $$s\left(t\right)=s\left(0\right)\mathrm{e}\mathrm{x}\mathrm{p}\left(\sigma BH\left(t\right)+Lt-\frac{1}{2}{\sigma }^{2}{t}^{2H}\right), t\ge 0$$

The mean satisfies:

20 $$E\left(s\left(t\right)\right)={s}_{0}{e}^{Lt}, t\ge 0$$

Taking the derivative with respect to position gives the velocity expression:

21 $$v=\frac{ds}{dt}=sL+\sigma s\frac{dBH(\mathrm{t})}{dt}, s\left(0\right)={s}_{0}>0$$

In the formula, $\frac{dBH(\mathrm{t})}{dt}$ represents fractal white noise. In this paper, they are modeled as exponentially decaying random terms to simplify calculations.

22 $$v=\frac{a\sigma }{t+2}{e}^{{\sigma}_{1}-{\sigma}_{2}t}{f}_{random}\left(\alpha ,\beta \right)+bL$$

## Adaptive parameter calculation

The fluctuation parameters *σ*,${\sigma}_{1}$,${\sigma}_{2}$ are determined adaptively based on the population’s evolutionary state.

23 $$\left\{ {\begin{array}{*{20}l} {\sigma = \sqrt {\frac{1}{D}\mathop \sum \limits_{i = 1}^{D} \left( {s_{igbest} - s_{igworst} } \right)^{2} } } \hfill \\ {\sigma_{1} = \sigma f_{{{\mathrm{random}}}} \left( {0.2,1.0} \right)} \hfill \\ {\sigma_{2} = \sigma f_{{{\mathrm{random}}}} \left( {0.05,0.5} \right)} \hfill \\ \end{array} } \right.$$

where ${s}_{igbest}$ and ${s}_{igworst}$ represent the global optimal and global worst positions, respectively.

## Iteration rules for speed and position

Perform N iterations on the j-th dimension sub-vector:

24 $$\left\{ {\begin{array}{*{20}l} {v_{j}^{k} = \frac{a\sigma }{{k + 2}}e^{{\sigma_{1} - k\sigma_{2} }} f_{{{\mathrm{random}}}} \left( { - 0.5,0.5} \right) + bL_{j}^{k - 1} } \hfill \\ {a = 0.382,{ }b{ } = \sigma f_{{{\mathrm{random}}}} \left( {0,2.0} \right)} \hfill \\ \end{array} } \right.$$

Update location by accepting based on fitness score:

25 $$s_{j}^{k} = \left\{ {\begin{array}{*{20}l} {s_{j}^{k - 1} + v_{j}^{k} ,{ }\;\;{\mathrm{if}}\;\;{ }f\left( {s_{0}^{k} } \right) > f\left( {s_{{0^{T} }}^{k} } \right)} \hfill \\ {s_{j}^{k - 1} ,\;\;{\text{ if}}\;\;f\left( {s_{0}^{k} } \right) \le f\left( {s_{{0^{T} }}^{k} } \right)} \hfill \\ \end{array} } \right.$$

## Adaptive learning factor ${{\boldsymbol{L}}}_{{\boldsymbol{j}}}^{{\boldsymbol{k}}}$

The learning weight ${L}_{j}^{k}$ is updated in the following three-step manner (the decay factor *n*= 4.0, the error tolerance *\varepsilon*= 0.0001) :

26 $$s_{j}^{k} = \left\{ {\begin{array}{*{20}l} {s_{j}^{k - 1} + v_{j}^{k} ,{ }\;\;{\mathrm{if}}\;\;{ }f\left( {s_{0}^{k} } \right) > f\left( {s_{{0^{T} }}^{k} } \right)} \hfill \\ {s_{j}^{k - 1} ,\;\;{\text{ if}}\;\;f\left( {s_{0}^{k} } \right) \le f\left( {s_{{0^{T} }}^{k} } \right)} \hfill \\ \end{array} } \right.$$

## References

[1] Ma, X., Mao, C. & Liu, G. Can robots replace human beings?—assessment on the developmental potential of construction robot. *J. Build. Eng.* **56**, 104727. 10.1016/j.jobe.2022.104727 (2022).

[2] Pham, P. A. H. & Hoang, N. D. Metaheuristic optimization of extreme gradient boosting machine for enhanced prediction of lateral strength of reinforced concrete columns under cyclic loadings. *Results Eng.* **24**, 103125. 10.1016/j.rineng.2024.103125 (2024).

[3] Hoang, N. D., Pham, P. A. H., Huynh, T. C., Cao, M. T. & Bui, D. T. Geospatial urban heat mapping with interpretable machine learning and deep learning: A case study in Hue City Vietnam. *Earth Sci. Inf.* **18**, 64. 10.1007/s12145-024-01582-2 (2025).

[4] Rahmani, M. C. et al. Mechanics-based deep learning framework for predicting deflection of functionally graded composite plates using an enhanced whale optimization algorithm. *Math. Mech. Solids* 10.1177/10812865251398866 (2026).

[5] Brahim, A. O. et al. Prediction in percentage variation of frequencies on damaged pipe repaired by composite materials GFRP. *CFRP, and BFRP, Int. J. Pressure Vessel Piping.* **222**, 105768. 10.1016/j.ijpvp.2026.105768 (2026).

[6] Chang, S., Siu, M. F. F., Li, H. & Luo, X. Evolution pathways of robotic technologies and applications in construction. *Adv. Eng. Inform.* **51**, 101529. 10.1016/j.aei.2022.101529 (2022).

[7] Bouabdallah, A., Benaissa, A., Bouabdallah, M. A., Malab, S. & Khatir, A. Development and performance evaluation of self-leveling sand concrete: Enhanced fluidity, mechanical strength, durability, and non-destructive analysis. *Constr. Build. Mater.* **468**, 140463. 10.1016/j.conbuildmat.2025.140463 (2025).

[8] Sánchez, E., Nájera, A. & Sotomayor, O. Numerical study of the viscoelastic mechanical response of polystyrene in the process of thermoforming through the generalized Maxwell model, *Materials Today*. *Proceedings.* **49**, 107–114. 10.1016/j.matpr.2021.07.480 (2022).

[9] Batt, G. S., Gibert, J. M. & Daqaq, M. Small strain vibration of a continuous, linearized viscoelastic rod of expanded polymer cushion material. *J. Sound Vib.* **349**, 330–347. 10.1016/j.jsv.2015.03.039 (2015).

[10] Ruimin, Y., Jianwen, D. & Feng, J. I. The constitutive of binary medium for soils based on Voigt model and Reuss model. *Procedia Earth Planet. Sci.* **5**, 218–221. 10.1016/j.proeps.2012.01.038 (2012).

[11] López-Almansa, F. & Kharazian, A. New formulation for estimating the damping parameter of the Kelvin-Voigt model for seismic pounding simulation. *Eng. Struct.* **175**, 284–295. 10.1016/j.engstruct.2018.08.024 (2018).

[12] Hu, X. & Gutierrez, M. Viscoelastic Burger’s model for tunnels supported with tangentially yielding liner. *J. Rock Mech. Geotech. Eng.* **15**(4), 826–837. 10.1016/j.jrmge.2022.07.013 (2023).

[13] Jiao, D. & De Schutter, G. Insights into the viscoelastic properties of cement paste based on SAOS technique. *Constr. Build. Mater.* **357**, 129320. 10.1016/j.conbuildmat.2022.129320 (2022).

[14] Binder, E. et al. Thermally activated viscoelasticity of cement paste: minute-long creep tests and micromechanical link to molecular properties. *Cem. Concr. Res.* **163**, 107014. 10.1016/j.cemconres.2022.107014 (2023).

[15] Zeng, K., Stauff, N. E., Hou, J. & Kim, T. K. Development of multi-objective core optimization framework and application to sodium-cooled fast test reactors. *Prog. Nucl. Energy* **120**, 103184. 10.1016/j.pnucene.2019.103184 (2020).

[16] Kempf, S., Forget, B. & Hu, L. W. Kriging-based algorithm for nuclear reactor neutronic design optimization. *Nucl. Eng. Des.* **247**, 248–253. 10.1016/j.nucengdes.2012.03.001 (2012).

[17] Kim, K. Y. & Lee, S. M. Shape optimization of inlet plenum in a PBMR-type gas-cooled nuclear reactor. *J. Nucl. Sci. Technol.* **46**(7), 649–652. 10.1080/18811248.2007.9711571 (2009).

[18] Huang, T. T. & Lee, T. T. Constructing response surfaces with Kriging models for quick estimation of radionuclide activity in spent nuclear fuel. *Appl. Radiat. Isot.* **222**, 111858. 10.1016/j.apradiso.2025.111858 (2025). 40300273

[19] Zhou, J., Qian, S., Han, T., Zhang, R. & Ren, J. Error prediction for machining thin-walled blade with Kriging model. *Result. Eng.* **26**, 104645. 10.1016/j.rineng.2025.104645 (2025).

[20] Li, X., Zhao, H., He, X. & Ding, H. A novel cartesian trajectory planning method by using triple NURBS curves for industrial robots. *Robot. Comput. Integrat. Manuf.* **83**, 102576. 10.1016/j.rcim.2023.102576 (2023).

[21] MacMillan, W. R., Irani, R. A. & Ahmadi, M. Planar image-space trajectory planning algorithm for contour following in robotic machining. *CIRP J. Manuf. Sci. Technol.* **42**, 1–11. 10.1016/j.cirpj.2023.01.005 (2023).

[22] Ekrem, Ö. & Aksoy, B. Trajectory planning for a 6-axis robotic arm with particle swarm optimization algorithm. *Eng. Appl. Artif. Intell.* **122**, 106099. 10.1016/j.engappai.2023.106099 (2023).

[23] Wang, R., Zhao, N. & Dong, Y. Multi-objective trajectory planning and implementation of a metamorphic palletizing robot. *Proc. Inst. Mech. Eng. C J. Mech. Eng. Sci.* **238**(20), 10091–10106. 10.1177/09544062241263411 (2024).

[24] Khatir, A. et al. A new hybrid PSO-YUKI for double cracks identification in CFRP cantilever beam. *Compos. Struct.* **311**, 116803. 10.1016/j.compstruct.2023.116803 (2023).

[25] Khatir, A. et al. An efficient improved gradient boosting for strain prediction in near-surface mounted fiber-reinforced polymer strengthened reinforced concrete beam. *Front. Struct. Civ. Eng.* **18**(8), 1148–1168. 10.1007/s11709-024-1079-x (2024).

[26] Yagüe, M. P., García, J. E. S. & Penas, M. S. Human-intelligent trajectory optimization for robotic manipulators with hybrid PSO-PS algorithm. *Adv. Eng. Inform.* **69**, 103941. 10.1016/j.aei.2025.103941 (2026).

[27] Elgohr, A. T., Khater, H. A. & Mousa, M. A. Trajectory optimization for 6 DOF robotic arm using WOA, GA, and novel WGA techniques. *Results Eng.* **25**, 104511. 10.1016/j.rineng.2025.104511 (2025).

[28] Sun, Z., Voigt, T. & Shah, S. P. Rheometric and ultrasonic investigations of viscoelastic properties of fresh Portland cement pastes. *Cem. Concr. Res.* **36**(2), 278–287. 10.1016/j.cemconres.2005.08.007 (2006).

[29] Qiu, X., Qiu, X. & Liao, F. A fractal evolutionary particle swarm optimizer. *J. Comput.* **8**(5), 1303–1308. 10.4304/jcp.8.5.1303-1308 (2013).

[30] Jeong, S., Murayama, M. & Yamamoto, K. Efficient optimization design method using kriging model. *J. Aircr.* **42**(2), 413–420. 10.2514/1.6386 (2005).

[31] Jiaqiang, E. et al. Orthogonal experimental design of liquid-cooling structure on the cooling effect of a liquid-cooled battery thermal management system. *Appl. Therm. Eng.* **132**, 508–520. 10.1016/j.applthermaleng.2017.12.115 (2018).

[32] Liu, J. et al. TS-REPLICA: A novel replica placement algorithm based on the entropy weight TOPSIS method in spark for multimedia data analysis. *Inf. Sci.* **626**, 133–148. 10.1016/j.ins.2023.01.049 (2023).

[33] Çiftçioğlu, A. Ö., Delikanlı, A., Shafighfard, T. & Bagherzadeh, F. Machine learning based shear strength prediction in reinforced concrete beams using Levy flight enhanced decision trees. *Sci. Rep.* **15**(1), 27488. 10.1038/s41598-025-12359-y (2025). 40721452 PMC12304224

[34] Shafighfard, T., Asgarkhani, N., Kazemi, F. & Yoo, D. Y. Transfer learning on stacked machine-learning model for predicting pull-out behavior of steel fibers from concrete. *Eng. Appl. Artif. Intell.* **158**, 111533. 10.1016/j.engappai.2025.111533 (2025).

[35] Kazemi, F., Shafighfard, T., Jankowski, R. & Yoo, D. Y. Active learning on stacked machine learning techniques for predicting compressive strength of alkali-activated ultra-high-performance concrete. *Arch. Civ. Mech. Eng.* **25**(1), 24. 10.1007/s43452-024-01067-5 (2024).

[36] Bagherzadeh, F. & Shafighfard, T. Ensemble machine learning approach for evaluating the material characterization of carbon nanotube-reinforced cementitious composites. *Case Stud. Constr. Mater.* **17**, e01537. 10.1016/j.cscm.2022.e01537 (2022).

[37] Deb, K., Agrawal, S., Pratap, A., Meyarivan, T. A fast elitist non-dominated sorting genetic algorithm for multi-objective optimization: NSGA-II, In: *International Conference on Parallel Problem Solving from Nature*, 2000, pp. 18–20. 10.1007/3-540-45356-3_83

[38] Lacevic, B. & Amaldi, E. Ectropy of diversity measures for populations in Euclidean space. *Inf. Sci.* **181**(11), 2316–2339. 10.1016/j.ins.2010.12.004 (2011).

[39] Zheng, K., Yang, R. J., Xu, H. & Hu, J. A new distribution metric for comparing Pareto optimal solutions. *Struct. Multidiscip. Optim.* **55**, 53–62. 10.1007/s00158-016-1469-3 (2017).

[40] Zitzler, E., Thiele, L., Laumanns, M., Fonseca, C. M. & Da Fonseca, V. G. Performance assessment of multiobjective optimizers: An analysis and review. *IEEE Trans. Evol. Comput.* **7**(2), 117–132. 10.1109/TEVC.2003.810758 (2003).

[41] Li, M., Yang, S. & Liu, X. Shift-based density estimation for Pareto-based algorithms in many-objective optimization. *IEEE Trans. Evol. Comput.* **18**(3), 348–365. 10.1109/TEVC.2013.2262178 (2013).

